# Limited tree mortality in unburned areas linked to bark beetle spillover from wildfires

**DOI:** 10.1002/eap.70066

**Published:** 2025-07-02

**Authors:** Robert A. Andrus, Joel Egan, Nathan Ivy, Laura Lowrey, Cameron E. Naficy, Brytten Steed, Arjan Meddens

**Affiliations:** ^1^ School of the Environment, Washington State University Pullman Washington USA; ^2^ USDA Forest Service, State & Private Forestry, Forest Health Monitoring Missoula Montana USA; ^3^ USDA Forest Service, State & Private Forestry, Forest Health Protection Medford Oregon USA; ^4^ Ecology Program and Forest Health Protection Pacific Northwest Region, USDA Forest Service Baker City Oregon USA; ^5^ Department of Forest Ecosystems and Society College of Forestry, Oregon State University Corvallis Oregon USA; ^6^ USDA Forest Service, State & Private Forestry, Forest Health Protection Missoula Montana USA

**Keywords:** climate change, *Dendroctonus pseudotsugae*, disturbance interactions, fire ecology, insect susceptibility models, Northern Rocky Mountains

## Abstract

Increased fire activity in the western United States since 2000 has produced an abundance of fire‐injured trees at risk to lethal attack by bark beetles. Large populations of bark beetles reproducing in fire‐injured trees may disperse (or spillover) from inside the fire perimeter to adjacent, unburned forests, potentially causing extensive tree mortality. In the western United States and Canada, fire‐injured Douglas‐fir (DF; *Pseudotsuga menziesii*) are frequently colonized by Douglas‐fir beetle (DFB; *Dendroctonus pseudotsugae*), prompting concern among land managers about elevated risk of spillover. We investigated spatiotemporal patterns of DF tree mortality from DFB in unburned areas surrounding 61 wildfires (2000–2017) with a high likelihood for spillover in the Northern Rocky Mountains, USA. We developed a multiple‐scale analytical framework to examine tree mortality potentially associated with spillover following fire. Synchronous fluctuation in the amount of DF mortality within and beyond the flight distance of DFB in the region and surrounding individual fires (0–10 km) suggested that DFB activity primarily responded to a broader scale process, such as drought, rather than proximity to burned trees. Using shorter and longer range dispersal scenarios, we estimated that at <0.25 km from the fire perimeter, the dominant source of DFBs transitioned from burned to unburned sources due to the closer proximity of DFBs from unburned sources. Some fires (8%–15%; range of fires from sensitivity analysis) did exhibit evidence of DFB spillover, but spillover occurred <1 km from fires (based on our criteria) and DF tree mortality associated with spillover was 0.2%–0.3% of total DF damage area during the study period. Spillover was not associated with climate conditions that increase host tree stress, rather it was associated with greater DF mortality from DFB in the prior year in the same area (i.e., poorly linked to spillover). Site‐specific monitoring of post‐fire DFB populations in susceptible, unburned DF forests adjacent to fires by land managers may be necessary to determine the risk of DFB emigrating from burned areas. Our findings inform post‐fire planning and the ecological implications of disturbance interactions that occurred in the early 21st century during a period of amplified wildfire and DFB activity.

## INTRODUCTION

Disturbances play an essential role in shaping vegetation structure and composition in forest ecosystems. Prior disturbances often influence subsequent disturbances (i.e., disturbance interactions) and have the capacity to drive rapid ecological change (Johnstone et al., 2016; Lindenmayer et al., 2010). A prior disturbance may amplify or dampen the occurrence, extent, or severity of the subsequent disturbance (i.e., linked disturbance interaction; Simard et al., 2011). For example, populations of some tree‐killing bark beetles' species may rapidly increase in wind‐felled trees and spread into neighboring forested areas (Christiansen & Bakke, 1988; Gandhi et al., 2007). Alternatively, minimal influence of a prior disturbance on a subsequent disturbance (i.e., neutral interaction) may occur if both disturbances are responding to a common initiating process. For example, increased bark beetle and wildfire activity are both associated with warmer and drier conditions (Bentz et al., 2010; Juang et al., 2022), and bark beetle severity had minimal effects on subsequent fire severity in the Northern Rocky Mountains, USA, due to the stronger influences of extreme fire weather, climate, topography, and high fuel loads (Harvey et al., 2014). Regardless of the nature of the interaction, interacting disturbances may produce unexpected vegetation trajectories following the disturbance interaction (i.e., compound interactions; Paine et al., 1998). As the occurrence of climate‐sensitive disturbances increases with warming temperatures, land managers and ecologists need to understand the nature of disturbance interactions to address resource management challenges and maintain the socioeconomic benefits provided by forests, such as ecosystem services and timber production (Burton et al., 2020; Vose et al., 2018).

The increase in area burned in the western United States since c. 2000 has produced an abundance of fire‐injured trees at risk to infestation by bark beetles (Hood & Bentz, 2007; Juang et al., 2022), and burned areas have been proposed as initiating locations for outbreaks of bark beetles. Subcortical bark beetles feed on and reproduce in the phloem of host conifer species. Most of the time, beetles are present at low densities across the landscape (i.e., their endemic phase) and restricted to colonizing weakened or damaged host material. During the endemic phase, populations are primarily regulated by the negative density‐dependent effects of tree defenses. If abundant weakened and damaged host material is available, some bark beetle species can exhibit positive feedbacks that rapidly increase or sustain large populations (i.e., their epidemic phase) as a result of density‐dependent changes in host utilization (Berryman, 1987; Raffa et al., 2008). Bark beetles use aggregation pheromones to coordinate mass attacks on individual trees that may sufficiently compromise tree physiological processes, such as the flow of water and nutrients, and cause tree mortality. Key biophysical conditions for the development of bark beetle epidemics include (1) susceptible tree and stand characteristics, such as high abundances of large‐diameter trees injured by fire; (2) dry and/or warm conditions that elevate host tree physiological stress; and (3) warm temperatures that increase winter survival and development rates (Bentz et al., 2010; Jaime et al., 2024; Raffa et al., 2015). Bark beetle populations collapse when these conditions are no longer met. In the context of bark beetle population dynamics in burned areas, low to moderate fire injury reduces the physiological capacity of trees to defend against bark beetle attack and produces a pulse of nutritional resources and suitable breeding material for several species of bark beetle in their preferred host tree species (Cunningham et al., 2005; Davis et al., 2012; McHugh et al., 2003; Powell et al., 2012; Valor et al., 2021). When bark beetles are present in areas with an abundance of fire‐injured trees, bark beetle populations may grow rapidly and disperse into unburned forests (i.e., spillover) to reduce intraspecific competition in burned areas (density‐dependent dispersal; Berryman, 1987), potentially resulting in extensive tree mortality in unburned areas.

In unburned forests adjacent to burned areas, bark beetle populations are influenced by reproduction, mortality, and migrations from burned and unburned areas (Powell et al., 2012). In this context, tree mortality from bark beetles in unburned areas outside of fire perimeters can be explained by three, non‐mutually exclusive hypotheses. First, large populations of bark beetles originating from fire‐injured trees may spread into unburned areas and successfully mass‐attack green trees (spillover hypothesis). High rates of tree mortality in unburned areas in proximity (spatially clustered) to burned areas and within the typical flight distance of bark beetles are supporting evidence for the spillover hypothesis. Second, bark beetles emigrating from fire‐injured trees in burned areas may only successfully mass‐attack and kill trees in unburned areas during periods of elevated tree physiological stress (stress‐spillover hypothesis). Physiological stress may result from unfavorable climate conditions (e.g., warm temperatures, drought) that increase tree host stress and improve bark beetle fitness (e.g., development rates, winter survival), competition from neighboring trees, and/or another disturbance (Agne et al., 2018; Bentz et al., 2010; Wright et al., 1984). For example, defoliator activity increases susceptibility to bark beetle attack in some insect‐host systems (Hadley & Veblen, 1993; Wallin & Raffa, 1999). Third, tree mortality from bark beetles in unburned areas may be explained by in situ bark beetle populations responding to shifts in abiotic (e.g., climate) and biotic conditions (e.g., suitable hosts), with minimal emigration of bark beetles from burned areas (local reproduction hypothesis; Howe et al., 2021). Synchronous tree mortality in unburned areas within and beyond the common flight distance of bark beetles would be evidence for the local reproduction hypothesis. In summary, hypotheses concerning the source of bark beetles (burned or unburned) associated with tree mortality in unburned areas can be evaluated by assessing the spatiotemporal progression of post‐fire tree mortality, the proximity of this mortality from fire, and post‐fire climate conditions.

Douglas‐fir (*Pseudotsuga menziesii* var. *glauca*, hereafter DF) is the most abundant and commercially valuable tree species in interior forests of the western United States (Stanke et al., 2021). Among biotic disturbance agents, Douglas‐fir beetle (*Dendroctonus pseudotsugae* Hopkins; hereafter, DFB) is the most damaging native agent for DF in the western United States (Furniss et al., 1977). From 1997 to 2023, nearly 200,000 ha of DF tree mortality was attributed to DFB in the western United States (Andrus et al., 2025). Small populations of DFB are present in many DF forests and can mass‐attack and kill larger DF trees (c. >35 cm diameter at breast height) with compromised tree host defenses (Aukema et al., 2016; Rudinsky & Vité, 1956; Shore et al., 1999). When critical abiotic and biotic thresholds are exceeded, DFB populations may grow rapidly and cause elevated levels of tree mortality across large forest landscapes (Furniss et al., 1979; Schmitz & Gibson, 1996; Wright et al., 1984). However, periods of population growth of DFB are short‐lived and rarely sustained by positive feedbacks due to the dependence on stressed DF trees with low resistance to attack and the ability of undamaged, healthy DF trees to defend themselves (Aukema et al., 2016). Following fire, DFB commonly infests fire‐injured DF trees 1–3 years post‐fire, with infestations documented up to 5 years post‐fire (Cunningham et al., 2005; Hood & Bentz, 2007; Weatherby et al., 2001). Elevated populations of bark beetles in insect traps and concern for increased DF tree mortality from DFB in unburned areas surrounding fires have prompted management actions over the last several decades. For example, efforts to mitigate bark beetle impacts occurred following the Yellowstone Fire in Wyoming (Rasmussen et al., 1996; Ryan & Amman, 1996), the Castle Rock Fire in southern Idaho (Lazarus, 2011), and the 2000 fires in the Bitterroot Mountains in western Montana (Bulaon, 2003). An evaluation of when, how far, and for how long elevated DFB populations originating from within burned areas affects trees in unburned areas is needed to support post‐fire planning by resource managers.

Our goal was to investigate the spatiotemporal progression of DF tree mortality from DFB in unburned areas surrounding wildfires (excluding unburned areas within fire perimeters) for evidence of DFB dispersing from burned to unburned areas. We created a spatial model of DF forest susceptibility to DFB and selected 61 wildfires from 2000 to 2017 with highly susceptible DF forests within and surrounding the fire perimeter (i.e., fires with a higher likelihood for DFB dispersal to unburned areas) in the Northern Rocky Mountains, USA (NRM). From broad to fine spatial scales, we evaluated our hypotheses using the spatiotemporal progression of mortality, the likely source of DFBs (from burned or unburned areas), and the inciting factors (local reproduction, fire, climate). The research objectives were addressed with spatial analysis (Objectives 1–4) and field data collection (Objective 5; see Table 1 for analyses and summary of evidence supporting each hypothesis). (1) At the regional scale (all study fires aggregated), we assessed whether annual DF tree mortality from DFB near fires was more correlated with the trend in total burned area of susceptible DF forest in the study fires (spillover hypotheses) or total DF tree mortality from DFB in the study area (local reproduction hypothesis). (2) Across all fires individually (spillover analysis), we used a null model of randomized fire locations to evaluate whether DF tree mortality from DFB was higher than expected near fires (spillover hypothesis) or similar with distance from fire (local reproduction hypothesis). (3) To determine the likely source of DFBs in new areas of DF mortality in unburned areas surrounding fires, we evaluated the relative strength of the DFB population pressure from burned and unburned sources and assessed the distance from fire at which the dominant source of DFBs transitioned from burned to unburned sources using shorter and longer range dispersal scenarios. (4) To identify individual fires with DFB spillover (spillover fires), we developed and applied a set of criteria and then assessed the distance of spillover and the dependence of spillover (spillover drivers) on biophysical factors influencing tree physiological stress (spillover‐stress hypothesis). (5) Site disturbance agents (e.g., root disease, blowdown) may support local reproduction of DFB populations, confounding the attribution of DFB spillover to the spread of DFB from burned areas. For individual patches of DF mortality from DFB (25 sites), we assessed the site for disturbance agents in the field. Our results inform post‐fire planning by estimating the frequency and distance of DFB spillover.

**TABLE 1:** Overview of hypotheses explaining Douglas‐fir (DF) tree mortality from Douglas‐fir beetle (DFB) in unburned areas surrounding fires. For each hypothesis, the objectives, a brief overview of the datasets and statistical methods, and evidence supporting each non‐mutually exclusive hypothesis and the associated figure are included. Damage area (DA) is the area of damage (killed) DF trees.

Hypothesis	Objective	Datasets and statistical method	Supporting evidence
Spillover^a^	Regional analysis (Obj. 1)	DF DA (percentage of susceptible DF forest damaged; IDS) less than and greater than 1 km from all fires correlated to burned area (correlations lagged 2–5 year to match timing of DFB dispersal)	Strong correlation between annual burned area of susceptible DF forest and DF DA near fire (<1 km) during period when DFBs are dispersing from fire (2–5 years post‐fire; Figure 2).
	Spillover analysis (Obj. 2)	DF DA in buffer areas surrounding fires (original and random locations) from 1 to 6 years post‐fire (LMM)	Greater DF mortality from DFB near fire (<0.5 or >0.5–1.0 km) compared to areas further away (>1.0 km) and expected (random fire locations; Figure 3).
	Population pressure (Obj. 3)	DFB population pressure (distance weighted raster) from burned and unburned sources (GAM)	Greater DFB population pressure from burned than unburned sources (Figure 4).
	Spillover fires (Obj. 4)	DF DA in buffers surrounding fires (original locations only) from 2 to 5 years post‐fire	Meets criteria for spillover^b^ (Figure 5).
	Site disturbance agents (Obj. 5)	Field data assessment	No site level disturbance agents that would confound attribution of DFBs emigrating from burned areas (Figure 6).
Stress‐spillover^c^	Spillover drivers (Obj. 4)	Spillover fires predicted by bioclimatic variables (GLMM)	DFB spillover events associated with climate conditions indicative of tree physiological stress.
Local reproduction^d^	Regional analysis (Obj. 1)	See above	Strong correlation between annual DF DA near fire (<1 km) and further away (>1 km but within study area; Figure 2).
	Spillover (Obj. 2)	See above	Lower or average DF DA near fire (<0.5 or >0.5–1.0 km) compared to further away (>1 km) or expected (random fire locations; Figure 3).
	Population pressure (Obj. 3)	See above	Greater DFB population pressure from unburned than burned sources (Figure 4).
	Spillover fires (Obj. 4)	See above	Does not meet criteria for spillover^b^ (Figure 5).
	Site disturbance agents (Obj. 5)	See above	Site level disturbance agents associated with local DFB populations that confound attribution of DFBs emigrating from burned areas (Figure 6).

*Note*: Spatial datasets include insect and disease surveys (IDS), fire perimeters, and susceptible DF forest. The non‐spatial dataset was field data.

Abbreviations: GAM, generalized additive model; GLMM, generalized linear mixed model; LMM, linear mixed model.

^a^ Large populations of bark beetles originating from fire‐injured trees spread into unburned areas, resulting in high levels of tree mortality.

^b^ Fires with spillover to the buffer area <1.0 km from fire met the following criteria: damage area (DA, as percentage of susceptible DF forest) for the buffer area nearest the fire was (1) ≥90th percentile compared to buffer areas further away, (2) >1% to remove low levels of DF mortality (i.e., background mortality), and (3) greater than the ecoregional average for the respective year (see Figure 1 for ecoregions). Additionally, (4) the source of DFB population pressure was from burned areas only or mixed sources (unburned/burned).

^c^ Bark beetles emigrating from fire‐injured trees in burned areas may only successfully mass‐attack and kill trees in unburned areas during periods of elevated tree physiological stress.

^d^ In situ bark beetle populations in unburned areas increase (and cause mortality) in response to shifts in abiotic (e.g., climate) and biotic conditions (e.g., suitable hosts), with minimal emigration of bark beetles from burned areas.

## METHODS

### Study area

**FIGURE 1:**
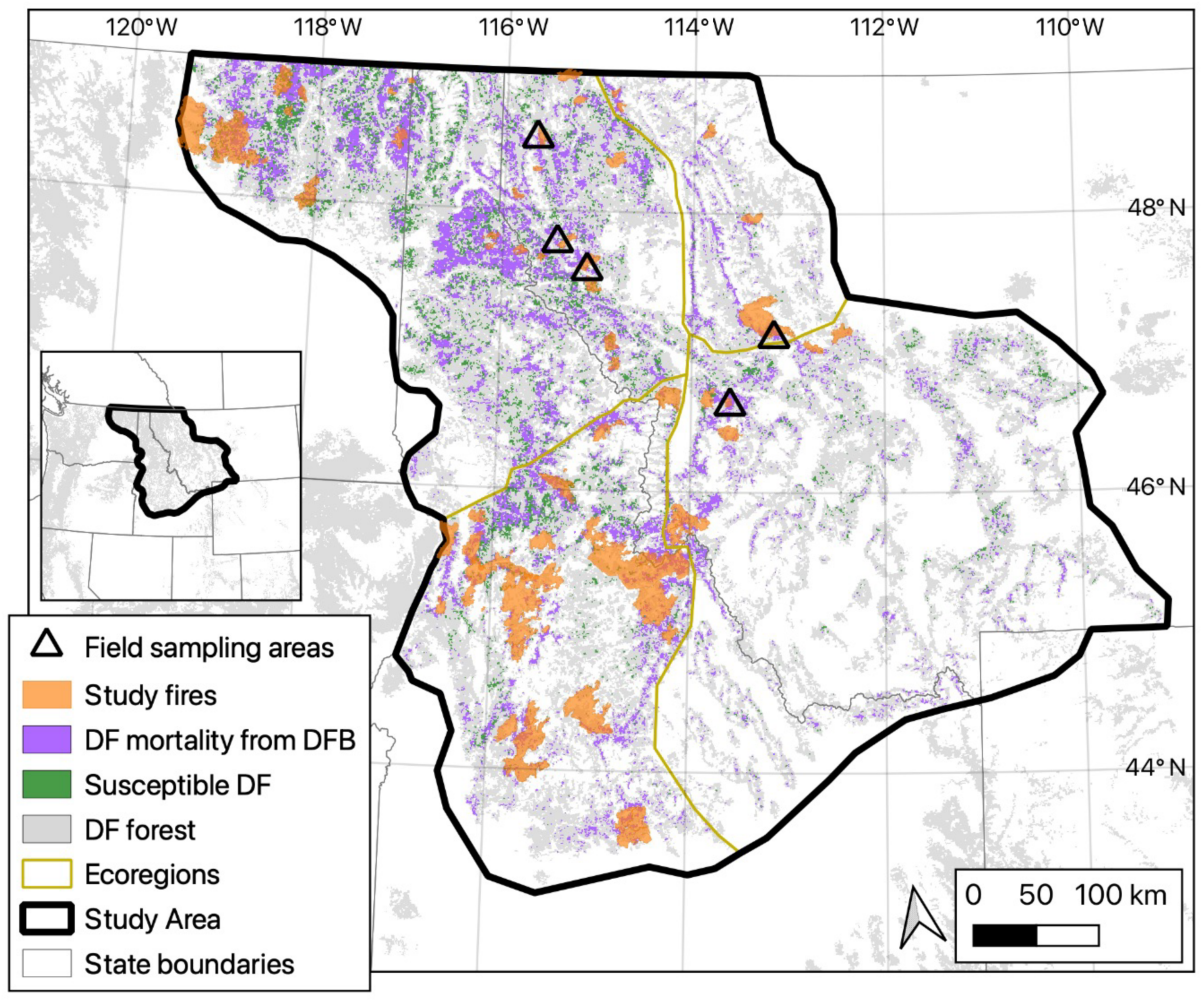
Map of study area with locations of field sampling areas (5 plots per triangle), areas burned in the 61 study fires from 2000 to 2017 (orange; MTBS, 2023), Douglas‐fir (DF) mortality from Douglas‐fir beetle (DFB) from 2000 to 2021 (purple; FHP, 2023), Random Forest‐predicted DF forest susceptible to DFB (green) (see Spatial model of DF forest susceptibility to DFB in *Methods*), DF forest distribution (gray; basal area >1 m^2^ ha^−1^; Krist et al., 2014), ecoregions (yellow lines; modified from US EPA, 2010), and the extent of the study area in the Northern Rocky Mountains (thick black line). The inset locates the study area within the western United States. The study fires burned 10,751.7 km^2^ of susceptible DF forest (all severities).

The study area was c. 150,000 km^2^ of DF forests in the NRM (Figure 1). In the NRM, summers are typically hot and dry, while winters are cold and snowy, but seasonal temperatures and precipitation regimes vary considerably across the study area (Halofsky et al., 2018). From 1895 to 2020, the mean annual air temperature increased 0.19–0.28°C per decade in the portion of the study area in Montana, with the greatest increases in winter (0.21–0.47°C) and spring (0.27–0.34°C) (Whitlock et al., 2017). From 1950 to 2015, total annual precipitation was relatively unchanged (Whitlock et al., 2017), but from 2000 to 2021, water year precipitation decreased (Williams et al., 2022). DF forests in the NRM are managed by federal agencies (e.g., US Forest Service), state agencies (e.g., State of Montana), and private landowners (e.g., timber companies). Within 5 km of the study fire perimeters, 93% of lands are managed by the US Forest Service.

Douglas‐fir is found in lower elevation dry to mesic mixed conifer forests and monotypic forests. The structure and composition of DF forests vary with topography, moisture availability, and disturbance history. At the lowest elevations and in xeric sites, DF grows primarily with ponderosa pine (*Pinus ponderosa*) or in monotypic stands on cooler and drier sites, especially in the southern portion of the study area where ponderosa pine is less common. At higher elevations and in mesic sites, DF grows with lodgepole pine (*Pinus contorta*), grand fir (*Abies grandis*), and western larch (*Larix occidentalis*) depending on geography.

Activity of biotic agents and wildfire are integral components of the disturbance regime in DF forests and influence DF abundance in the NRM (Halofsky et al., 2018; Stanke et al., 2021). Biotic agents, such as root disease and bark beetles, generally cause discontinuous patches of high mortality in localized areas (<1 ha) that can span across large, susceptible landscapes. By contrast, individual wildfires result in variable burn severity (tree mortality) over 10s to 100,000s of hectares (Hood et al., 2021). Activities associated with settlement of the NRM since the late 1800s have also transformed the structure and composition of many DF forests with implications for DFB and fire activity. For example, the exclusion of fires from many DF forests in the last century and cutting of large‐diameter trees (more fire resistant) from many lower elevation forests coincided with a cooler and wetter period (1950 to late 1990s) that allowed establishment of many new trees. Consequently, lower elevation forests have shifted from earlier‐seral, shade‐intolerant species (ponderosa pine and western larch) to late‐seral, shade‐tolerant tree species (e.g., grand fir and DF) and from larger diameter trees to primarily smaller to medium‐sized shade‐tolerant trees (Hessburg et al., 2000; Naficy et al., 2010). However, medium to larger diameter DF increased in relative density from 2001 to 2018 (Stanke et al., 2021), and the majority (~70%) of forest landscapes in our study area are currently susceptible to DFB (Krist et al., 2014). Since c. 2000, the shift to notably drier and warmer conditions is associated with increased fire and bark beetle activity (Andrus et al., 2025; Juang et al., 2022), highlighting the need to understand how wildfires may influence bark beetle activity.

### Data and data processing

We acquired spatial data for the following (see Appendix S1: Table S2 for summary): insect activity estimated from aerial surveys (FHP, 2023), spatial extent of fires (i.e., fire perimeters; MTBS, 2023), multiple stand structure and tree composition metrics (e.g., basal area) in 2002 (Krist et al., 2014), percent forest cover in 2000 (Hansen et al., 2013), forest management activities (USDA, 2022), and climate (PRISM, 2023).

To identify areas with DF tree mortality attributed to DFB, we downloaded annual spatial data of tree insect and disease detection surveys from 1998 to 2022 from the US Forest Service's National Insect and Disease Aerial Survey (IDS) program (FHP, 2023). The IDS is the most spatially extensive and best available dataset characterizing biotic disturbance agents (Coleman et al., 2018). The IDS is produced by trained observers in fixed‐wing aircraft that map areas (polygons) of insect and disease damage (i.e., airborne sketch mapping) and attribute damage to specific biotic agents and tree host species. Fire perimeters are a useful spatial reference point for mapping insect damage, suggesting accurate detection of insect damage surrounding burned areas. The timing of agent attack in IDS is based on foliage color change (green to red), which for DFB lags approximately 1 year behind the year of attack, but color change and foliage loss rate vary with moisture and temperature in the season following the attack (Belluschi & Johnson, 1969). Consequently, we subtracted 1 year from the survey year to estimate the year of DFB infestation. In general, the spatial accuracy of IDS polygons has been documented at 68% within 50 m and 79% within 500 m (Johnson & Ross, 2008), the host tree species attribution accuracy is 88% for DF in California, USA (Coleman et al., 2018), and the insect species attribution accuracy is 95% for DFB in California, USA (Coleman et al., 2018).

To estimate the canopy cover of DF mortality from DFB, we selected damage area (DA) as opposed to affected area (polygon area). DA excludes the mapped area of live trees which are often included to facilitate aerial mapping of discontinuous tree mortality (Egan et al., 2020). The accuracy of IDS data collected locally in the NRM and methods for estimating DA were both validated to be reasonable and slightly conservative estimates when compared to field and remotely sensed estimates of bark beetle‐caused canopy cover loss (e.g., ~82% of hydrologic units [i.e., HUC] correctly classified; Bright et al., 2020; Egan et al., 2020). To match the spatial resolution of the stand structure and composition metrics, we converted vector IDS data to annual rasters of DA with a resolution of 240 m for our analyses (Contiguous USA Albers Equal Area Conic projection). To account for the potential effects of prior defoliation on DFB‐caused mortality, IDS polygons for two defoliators of DF that are known to increase the risk of DFB attack (Cole et al., 2022; Howe et al., 2024; Wright et al., 1984), DF tussock moth (*Orgyia pseudotsugata*, *McDunnough*) and western spruce budworm (*Choristoneura freemani Razowski*), were converted to annual rasters (240 m) of presence and absence of defoliator activity.

### Field methods

To assess the mortality agents associated with DF mortality in IDS polygons, we sampled 25 field plots (total) in IDS polygons that were located <1.5 km from one fire that burned in 2016 or four fires that burned in 2017 (five plots and up to five IDS polygons per fire). Douglas‐fir beetles originating from fire‐injured trees are not likely to affect green trees in unburned areas outside of the fire perimeter until 2 years post‐fire as the DFB lifecycle generally requires 1 year to complete (eggs to adults; Schmitz & Gibson, 1996) and DFB preferentially colonize fire‐injured hosts. As such, we selected IDS polygons infested 2–6 years post‐fire (Hood & Bentz, 2007; Weatherby et al., 2001). Additionally, DF mortality from DFB and other biotic agents often occurs in patches of trees within a matrix of live DF and other tree species (McMillin & Allen, 2003), and IDS often lump small patches of mortality into one polygon (Johnson & Ross, 2008). Consequently, we located a patch of >5 trees within each IDS polygon that had died within the last 3 years (presence of red needles or >90% of fine‐twigs; Rasmussen et al., 1996) using aerial imagery and field reconnaissance. We established a 15‐m radius plot (0.07 ha) at the center of each patch to measure tree‐ and stand‐scale attributes and assess mortality agents associated with DF mortality. For each tree (>13 cm diameter at breast height) in the plot, we recorded the following: tree species, diameter at breast height (cm), tree status (live or dead), percent red and green needles, and external evidence of DFB activity (e.g., frass, entrance and exit holes). For dead DF trees only (no green needles), we removed two sections of bark (20 cm tall by 20 cm wide) at breast height to examine the inner bark for DFB and secondary beetle reproductive galleries. Secondary beetles included DF pole beetle (*Pseudohylesinus nebulosus*), DF engraver beetles (*Scolytus* spp.), and wood boring beetles (e.g., *Phaenops drummondi* and *Buprestis lyrata*) (Furniss & Johnson, 2002). We estimated the total proportion of the panel attacked by each insect species/group and averaged the two panels for each tree. Even though we observed substantial evidence of DFB, sampling likely underestimated DFB activity in the phloem panels as DFBs preferentially attack portions of the tree bole higher than ~3.6 m (Furniss, 1962).

To identify factors that may confound attribution of DF mortality to DFB spread from fire (Objective 5), we surveyed areas within 75 m of plot center for evidence of recent disturbance that may elevate localized populations of DFB. Disturbances included log decks, blown down DF trees with evidence of DFB that were of similar time since mortality as standing dead DF trees with DFB (last 5 years only), fire (spot fires or undetected fires), and root disease (e.g., *Armillaria*; Hagle et al., 2003).

### Analysis

#### Attribution accuracy of IDS polygons to DF mortality from DFB

Our goal was to confirm that DF tree mortality from DFB was a common source of mortality within the patches of dead trees mapped by IDS. In the 25 of 27 sites with DF mortality (two sites were misclassified and excluded), we calculated the percent of dead trees from the last c. 5 years that were DF trees with DFB galleries and the phloem occupancy of subcortical insects.

#### Spatial model of DF forest susceptibility to DFB

To select study fires and account for differences in the amount of DF forest susceptible to DFB surrounding the study fires, we created a spatially contiguous model of DF forest susceptible to DFB across multiple land ownerships (e.g., private, state, and federal) in the NRM. Following the methods of Tutland et al. (2023), we mapped DF stand susceptibility to DFB infestation in 2002 (earliest available reference date for spatial datasets) using Random Forest (RF) classification models (Breiman, 2001) to account for non‐linear relationships and correlated predictor variables (Cutler et al., 2007). The response variable was presence or absence of DFB DA within a grid cell from 1998 to 2022 (240 m raster; see *Data and data processing*) and included grid cells with low to high levels of damage resulting from varying levels of DFB populations (i.e., endemic to epidemic). Stand composition and structure metrics are commonly used to describe the susceptibility of a stand to bark beetle infestation, such as in stand hazard ratings (Randall et al., 2019). In our model, the predictor variables were DF basal area, stand basal area as DF (%), and stand density index (SDI) and forest cover (%) for all trees at a 240‐m resolution (see Appendix S1: Table S2 for details on each dataset; Hansen et al., 2013; Krist et al., 2014). Climate normal predictor variables were tested in preliminary models but were excluded from the final model because they did not improve model performance. The data for each variable in the RF model was extracted from points at the center of a 1.44‐km grid within areas of DF forest (>1 m^2^ ha^−1^; Krist et al., 2014) to reduce the effect of spatial autocorrelation. The data were split into a training (70% or 28,100 grid cells) and test dataset (30% or 12,044 grid cells). All predictors were included in the final model. To assess model performance and account for spatial autocorrelation, we compared predictions to withheld testing data using 10‐fold spatial cross‐validation to examine the following metrics: area under the receiver operating curve (AUC), sensitivity (rate of accurately predicted beetle presence), and specificity (rate of accurately predicted beetle absence). Models were built in R (R version 4.2.3; “ranger” and “tidymodels” packages; R Core Team, 2023; Wickham et al., 2019; Wright & Ziegler, 2017), and hyperparameters were tuned with spatial cross‐validation (“spatialsample” and “tune” packages; Kuhn, 2021; Silge & Mahoney, 2022).

To map DF forest susceptibility to DFB, we made probability predictions of DFB presence for grid cells with DF tree host presence (>1 m^2^ ha^−1^ host BA). Grid cells were defined as susceptible if the probability of infestation was ≥0.6 and not susceptible if probability of infestation was <0.6 (Appendix S1: Figures S1 and S2). The probability threshold was selected to maximize the true skill statistic (i.e., sensitivity + specificity – 1) and balance over/underestimation of the likelihood of DFB infestation (Appendix S1: Figures S1 and S2). We reclassified grid cells that were classified as “not susceptible” to “susceptible” if the cell experienced DFB infestation during the study period (7% of 1.5 million grid cells).

#### Selecting study fires

To include fires with a higher likelihood for DFB spread from burned to unburned areas, we first selected fires with >1000 ha of DF forest susceptible to DFB. Then, we selected fires with the following criteria: (1) fire year from 2000 to 2017 to allow for pre‐ and post‐fire insect surveys during the likely period of DFB colonization in burned and unburned areas, (2) >400 ha of DF forest susceptible to DFB in the area 500 m inside the fire perimeter to focus on fires with a higher likelihood for DFB population pressure on unburned areas, and (c) >600 ha of DF forest susceptible to DFB within 1 km of each fire (host availability) that was flown by IDS 5 of 6 years post‐fire to ensure sufficient sampling of unburned areas.

#### Spatial data extraction for the regional and spillover analyses

Correctly attributing DF mortality from DFB in unburned areas to DFB spread from fire‐injured trees is dependent on DFB's spatial (common flight distance) and temporal behavior (lifecycle). Mark and recapture studies suggest that 90% of DFB disperse less than 200 m and flight mills in laboratories estimate that the maximum flight distance is c. 5 km (Dodds et al., 2006; Jones et al., 2019). DFBs infest fire‐injured trees the year following fire and DFBs from fire‐injured trees may begin infesting unburned areas (i.e., spillover) as early as 2 years post‐fire, with a higher likelihood for infestation 3–5 years post‐fire. Initially infested unburned areas with DFBs from burned sources may be a source of DFBs for continued spread.

To assess the spatiotemporal progression in DF mortality from DFB (Objectives 1, 2, and 4), we extracted multiple forest and DA attributes from concentric buffer areas surrounding each study fire perimeter for each year from 1 to 7 years post‐fire (observed dataset). For comparison, we randomly shifted the perimeter of each study fire to 15 new locations (expected dataset) within 5–50 km of the original study fire that met the criteria used for study fire selection. For the fires in the observed and expected dataset, we extracted the following attributes from each buffer area: fire attributes (ID, name, area), IDS flown area (in hectares), IDS polygons of DF DA from DFB (in hectares), area of DF forest (in hectares), area of DF forest susceptible to DFB (in hectares), and area defoliated (in hectares; 10 years prior to fire) (see Appendix S1: Figure S3 for workflow). Buffer widths (in kilometers) were in 0.5‐km increments up to 10 km (see Appendix S1: Figure S4 for example), and the analysis was repeated using 1.0‐km buffer width areas to test the sensitivity of the results to buffer width. The 50 m nearest the fire was excluded from the buffer area to account for known spatial inaccuracies in fire perimeters and heat injury from the fire (Gannon et al., 2020). Buffers of 0.5 km accounted for spatial inaccuracies in aerial survey polygons, reduced splitting aerial survey polygons overlapping two buffers (DA split proportionally between buffers for overlapping polygons), and exceeded the population distance of most DFB each year. Areas not burned in the focal fire or experiencing forest management activities (compiled from multiple sources; see Table 1) from 5 years pre‐ and post‐focal fire were buffered by 0.5 km and removed from the analysis. Given the low levels of annual DF tree mortality (generally <1% of 240‐m grid cell), we did not adjust area of susceptible DF forest by prior DA. Data were analyzed in R with the “terra” package (Hijmans, 2023) and “tidyverse” package (Wickham et al., 2019).

#### Regional analysis of DF mortality from DFB and burned area

In the aggregated regional analysis (Objective 1), we totaled annual DF DA <1 km (hereafter, DA < 1 km) and >1 km from the study fires (hereafter, DA > 1 km) as well as burned area of DF forest susceptible to DFB by year. For DA < 1 km, we only included the period 2–5 years post‐fire to correspond to the likely timing of DFB spread to unburned areas. DA was standardized as a percent of susceptible DF forest within the respective area. A strong correlation (Spearman) between DA < 1 km and burned area of DF forest susceptible and a poor correlation between DA < 1 km and DA > 1 km would be supporting evidence for the spillover hypotheses, whereas the inverse is evidence for the local reproduction hypothesis and a regional driver of DFB activity (e.g., climate).

#### DF mortality from DFB with distance from and time since fire

For the spillover analysis (Objective 2), we performed two analyses using the observed and expected datasets that examined the spatiotemporal progression of DF tree mortality surrounding fires. Douglas‐fir DA clustered around the original fire location relative to further away would be evidence for DFB spillover. To test for differences in DA (percentage of susceptible forest) with time since and distance from fire, we used a linear mixed model (LMM) with buffer area distance from fire interacted with year as categorical predictor variables (fixed effects; Pinheiro et al., 2022). Buffer areas within fire were nested random effects. Damage area (%) was standardized (subtract mean and divide by SD) by fire to allow comparison across years. The LMM was followed by a pairwise test of means between years to detect increases in DA over time and differences in means between all buffer area pairs within year. We focused on pairwise comparisons between the two buffer areas nearest the fire perimeter (0–0.5 and 1.0‐km buffer area) where DFBs are most likely to spillover and buffer areas further away (>1.0 km; “emmeans” package in R; Lenth, 2023). We only corrected *p*‐values for the subset of pairwise tests (“Holm–Bonferroni” method).

To assess whether the original fire location was surrounded by greater than expected DF tree mortality from DFB spillover, we used an observed minus expected analysis. Specifically, we differenced DA (in percentage) in the observed dataset from DA (in percentage) in each of the 15 randomized fire locations (i.e., expected) to incorporate the variability into the analysis. The difference between the observed and expected DA (in percentage) was the response variable in an LMM, and categorical predictor variables (fixed effects) were buffer area distance from fire interacted with year. Buffer area within fire was nested random effects. The LMM was followed by pairwise comparisons (same methods as above).

#### Comparing DFB population pressure from burned and unburned sources

Douglas‐fir tree mortality surrounding fires in the years following fire are influenced by DFBs originating from burned and unburned areas. We compared estimates of the DFB population pressure (hereafter, DFB pressure) from burned and unburned sources (Objective 3) using IDS polygons mapped as DF mortality from DFB surrounding fire (0–2.5 km) and following fire (1–6 years). Douglas‐fir beetle pressure estimates the size and proximity of DFB populations from burned (spillover hypotheses) and unburned areas (local reproduction hypothesis) that can disperse and attack currently unaffected stands in unburned areas. Douglas‐fir beetle pressure from burned and unburned sources was estimated using different approaches based on data availability. Insect survey polygons are not or inconsistently surveyed in burned areas. For DFBs originating from burned areas, we applied a distance weighted raster (240‐m resolution to match other datasets) estimating DFB pressure (described below) to the DF forest susceptible to DFB (presence/absence raster) that burned (all severities) within each fire perimeter (see Appendix S1: Figure S5A for example). Our method likely overestimates DFB activity from burned areas for three reasons. (1) DFBs prefer low to moderate severity burned trees with moister phloem (Hood & Bentz, 2007), and we included all burn severity classes. (2) Only 15%–76% of fire‐injured DF trees are infested by DFB (Appendix S1: Table S1), and we assumed that DFBs infested entire grid cells with susceptible DF forest. (3) DFB activity is likely higher 3–5 years post‐fire, and we assumed constant DFB pressure from burned areas during the 2‐ to 6‐year post‐fire period. For DFBs originating from unburned areas, we applied the same distance weighted raster to DFB activity (presence/absence) in the two prior years to account for DFB populations likely persisting in areas with DF mortality (see Appendix S1: Figure S5B–E for example).

To estimate DFB pressure with distance from likely sources of DFB activity, we created distance weighted rasters for shorter and longer range dispersal scenarios to assess the sensitivity of the results. The shorter range dispersal scenario balanced primarily short‐range dispersal (<200 m or the common flight distance of DFB) while accounting for some longer distance dispersal (max. distance 720 m). For the shorter range dispersal scenario, the weights were assigned by distance as 1.0 for ≤240 m, 0.3 for >240 m to ≤480 m, and 0.1 for >480 m to ≤720 m. The longer range dispersal extended the dispersal distance to 1440 m, and the weights were 1.0 for ≤480 m, 0.3 for >480 m to ≤960 m, and 0.1 for >960 m to ≤1440 m. Our shorter range distance weights more closely corresponded to the common flight distances observed for DFBs (see above) and the recommendations of USDA Forest Health and Protection for the local zone of imminent risk, which is defined as the adjacent, susceptible DF forest within approximately 0.8 km of DFB populations in standing trees, log‐decked timber, and/or blowdown trees (Withrow et al., 2013).

For each IDS polygon in unburned areas, we extracted the DFB pressure weights from burned and unburned sources, the year infested (1–6 years post‐fire), and the Euclidean distance from fire to the polygon centroid (240 m raster). To account for DFB spread from burned to unburned areas and continued spread of DFBs from burned sources within unburned areas, we updated the distance weighted rasters to include sources of DFB from burned areas in unburned areas beginning 3 years after fire (i.e., 1 year after DFBs may begin spreading to unburned areas) if DFB pressure from burned sources exceeded unburned sources. For burned and unburned sources of DFB, we modeled DFB pressure with distance from fire for each fire individually using generalized additive models (GAM) to account for non‐linear relationships (R Core Team, 2023). The analysis was restricted to 35 fires with >20 IDS polygons to improve model performance. For each fire, we assessed the distance from fire at which the dominant source of DFB pressure switched from burned to unburned.

#### Identifying fires with spillover and drivers of spillover

To identify individual fires with evidence for spillover (Objective 4), we developed and applied a set of four criteria to the amount of DF tree mortality with time since and distance from fire for each year individually (observed dataset). Our criteria for spillover were purposefully conservative, designed to identify fires with strong evidence of spillover radiating away from burned areas in a linear progression, and cautiously interpreted to accommodate data uncertainty. Our criteria for spillover to the buffer area nearest the fire (<0.5 km from fire) were DF DA (as a percentage of susceptible DF forest damaged) in the buffer area nearest the fire was (1) ≥90th percentile compared to buffer areas further away, (2) >1% to remove low levels of DF mortality (i.e., background mortality), and (3) greater than the ecoregional average for the respective year (see Figure 1 for ecoregions). Additionally, (4) the source of DFB pressure was from burned areas only or mixed sources (unburned/burned). We selected the 90th percentile to reduce false detection of spillover and to identify locations with high populations of DFB that may re‐infest unburned areas adjacent to the fire or spread away from burned areas. Following the initial detection of DFB spillover, we adjusted our criteria to account for DFB activity that may repeatedly infest areas near fires or progress further away from burned areas in subsequent years. For only those fires with evidence for DFB spillover to the area nearest fire (e.g., <0.5 km), we added an additional buffer area further from burned areas (e.g., 0.5–1.0 km) for DFB to spread into during the following year. Additionally, we lowered the percentile threshold for detection of DFB spillover to the 80th percentile to account for the possibility of high levels of DFB infestation in multiple buffer areas. To account for the source of DFB pressure, we extracted the estimate of DFB population pressure from burned and unburned sources on individual IDS polygons within buffer areas with evidence of DFB spillover. Then, we tested whether DFB pressure (response variable) varied by source (burned or unburned) within buffer areas using a LMM with fire as a random effect (see *Methods*). The LMM was followed by a pairwise test of DFB sources (burned and unburned) by buffer area, and results were incorporated into the DF spillover mortality analysis.

To determine if physiological stress of DF trees was a prerequisite for DFB spillover (Objective 4: spillover drivers), we tested whether the presence (1) or absence (0) of DFB spillover was associated with biophysical variables indicative of tree stress and bark beetle survival (snow water equivalent, winter air temperature, and drought indices for spring, summer, and annual), availability of DFB breeding habitat (burned susceptible DF forest), and tree host availability (susceptible DF forest) within and surrounding focal fires (see Appendix S1: Table S5 for justification and expectations for biophysical predictors). For predictor variables that varied over time (e.g., climate), we used generalized linear mixed‐effects models (GLMM) with a binomial error distribution and fire as a random effect to account for repeated measures (Brooks et al., 2017). For predictor variables that did not vary over time (e.g., DF forest susceptible to DFB), we used generalized linear models with a binomial error distribution.

#### Site disturbance agents confounding attribution of DFB spread from fires

From tree assessments and descriptions of plots, we identified biotic agents associated with DF mortality and disturbance agents at the site that potentially support local reproduction of DFB within the stand and confound attribution of DF mortality in unburned forests to the spread of DFB from fire‐injured trees (Objective 5). Confounding factors (presence/absence in plot) included (1) root disease (source for injured trees; Schmitz & Gibson, 1996), (2) wind‐felled trees with DFB infestation of similar time since mortality (red needles and twigs) as recent (last 5 years) standing dead DF trees with DFB infestation, (3) DFB activity in the stand less than 5 years before fire, and (4) fire to account for unmapped spot fires. Douglas‐fir beetle activity prior to fire (3) was estimated based on the spatial overlap of aerial insect surveys from 2013 to 2017 and dead DF trees in field plots with evidence of DFB infestation, no red needles, and retention of most fine twigs.

## RESULTS

### Comparing IDS DF‐DFB polygons with field data

Within the patches of dead DF trees, 99% of all mortalities in the last ~5 years were DF trees (289 of 291). DFB was present on 96.2% of dead DF trees (278 of 289), while secondary beetles were found on 50.5% of dead DF trees. DFB occupied more phloem area (average 35.1%, range 0%–90%) than other subcortical insect species (average 6.3%, range 0%–55%; Appendix S1: Figure S6A), especially on larger diameter (> 29.5 cm diameter at breast height) DF trees (Appendix S1: Figure S6B,C).

### Spatial model of DF forest susceptibility to DFB

We predicted DF forest susceptibility to DFB infestation across the study area with stand structure and composition spatial datasets in a RF model (Figure 1). At a probability threshold of ≥0.60 in the RF model, 33% or 29,033 km^2^ of DF forest was susceptible to DFB (Figure 1, Appendix S1: Figure S2). The mean accuracy values of the RF model from 10‐fold spatial cross‐validation were 0.60 for AUC, 0.74 for sensitivity, and 0.39 for specificity. The 61 study fires included a wide range in area of DF forest susceptible to DFB from 0.5 km inside to 10 km outside the study fire perimeters (Appendix S1: Figure S7), indicating DF host availability for spillover.

### Regional analysis of burned area and DF mortality from DFB

**FIGURE 2:**
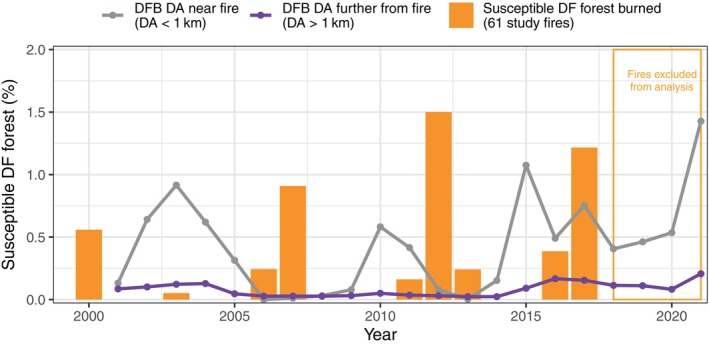
Percent of Douglas‐fir (DF) forest susceptible to Douglas‐fir beetle (DFB) that burned from 2000 to 2017 (orange) in 61 study fires (fires after 2017 excluded from analysis). Additionally, the DF damage area (DA, in percentage) near (<1.0 km) the study fires (gray; DA < 1 km) and further (>1.0 km) from the study fires (purple; DA > 1 km) from 2002 to 2021 as a percent of susceptible DF forest. DA < 1 km is for the period 2–5 years post‐fire to correspond with the likely timing of DFB spread to unburned areas outside of the fire perimeters. One year was subtracted from the acquisition year of DFB damage area to account for the 1‐year lag between DFB attack and foliage discoloration.

From 2002 to 2022, the total DF DA > 1 km (DA > 1.0 km from fire) was 94,860 ha and the annual area of susceptible DF forest that experienced DF mortality from DFB was <0.25% in the NRM (Figures 1 and 2). From 2 to 5 years after the 61 study fires, the DA < 1 km was 1990 ha, representing 2% of total DA during the study period in the NRM (DA < 1 km divided by DA > 1 km; Figure 2). The annual time series of DA < 1 km (in percentage) tracked the variability of DA > 1 km (in percentage), and they were highly correlated (Spearman's ρ = 0.782, *p* < 0.001). All years with high burned area of susceptible DF forest (2000, 2007, 2012, and 2017) were followed 3 years later by a peak in DA < 1 km and DA > 1 km. Correlations between burned area and DA < 1 km (lagged 2–6 years post‐fire) were highest 3 years post‐fire (ρ < 0.478, *p* > 0.162) and notably lower for all other lagged years (years 2, 4, 5, and 6; ρ < 0.197 and *p* > 0.561). Peaks in DF mortality from DFB (DA < 1 km and DA > 1 km; Figure 2) were often preceded by or occurred during years with warmer and drier than average climate conditions (Appendix S1: Figure S8). Similarly, fire years corresponded to years with warmer and drier than average conditions (Appendix S1: Figure S8).

### Spillover analysis of DF mortality from DFB with distance from and time since fire

**FIGURE 3:**
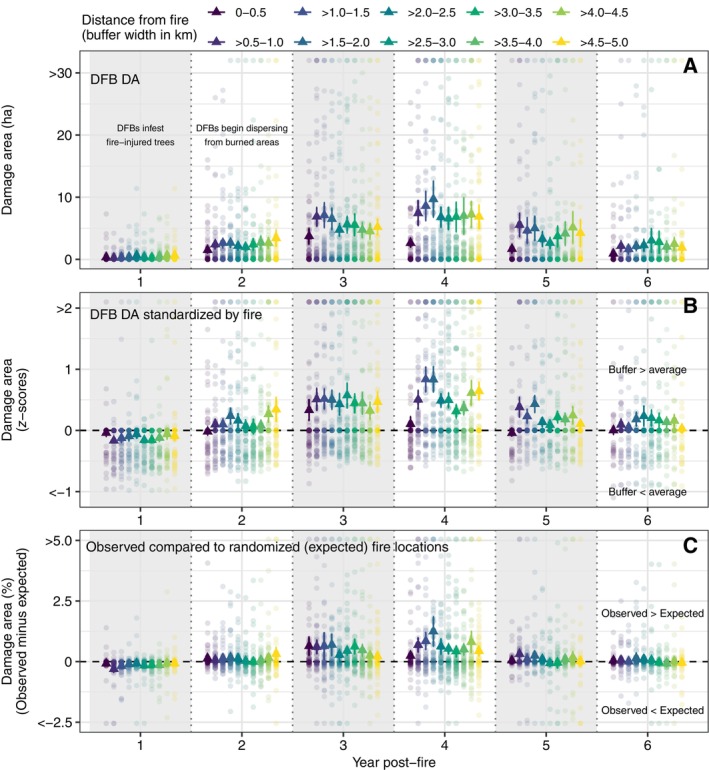
(A) Average Douglas‐fir (DF) damage area (DA) from Douglas‐fir beetle (DFB) from 1 to 6 years post‐fire for 0.5 km width buffer areas from 0 to 5 km from the fire perimeter in 61 fires in the Northern Rocky Mountains, USA, illustrating the amount and variability of DF DA surrounding fire perimeters. (B) Average DF DA with distance from fire (standardized with *z*‐scores by fire) to compare DA among buffer areas within year (i.e., spillover analysis; see Appendix S1: Figure S4 for example of buffer areas surrounding fire). The expected pattern in DA resulting from DFB spillover from fire‐injured trees to unburned areas (i.e., DFB spillover) would be greater DA immediately surrounding the fire perimeter (e.g., 0–0.5 km or >0.5–1.0 km buffer areas) relative to further away (e.g., >1 km). (C) Average of the observed DF DA (in percentage) minus the expected DA (in percentage) from 1 to 6 years post‐fire. Observed DA was subtracted from DA in each of 15 random fire locations and then averaged by buffer and year. The vertical lines are SEs, and the dots are values for individual fires.

Consistent with the regional analysis (Figure 2), DF DA was higher 3–4 years post‐fire relative to all other post‐fire years when considering the overall trend across all buffer areas (Figure 3A,B). Pairwise comparisons between years across all buffer areas confirmed that DA (standardized by fire) was significantly greater 3‐ and 4‐years post‐fire compared to all other years from 1 to 6 post‐fire (*p* < 0.05).

Comparing DF DA with distance from fire by year, DA was consistently lower near the fire (0–0.5 km buffer area) compared to areas slightly further away (e.g., >0.5–1.0, >1.0–1.5, >1.5–2 km) in years 2–5 post‐fire (Figure 3B). All pairwise comparisons of the two buffer areas nearest the fire (0–0.5 and >0.5–1.0 km) were not significantly different from areas further from fire (*p* > 0.05 after “Holm–Bonferroni” correction). In the fourth‐year post‐fire, DF DA was elevated in areas 1.0–2.0 km from fire compared to other distances from fire (potentially the result of long distance dispersal), but this difference was not statistically significant. For nearly all buffer areas, DF DA was highly variable among fires, particularly for the 2–5 years post‐fire (Figure 3A,B). Results were similar for buffer widths of 1 km (Appendix S1: Figure S9A,B).

The average difference between DA (%) in the buffer area near the observed fire locations (0–0.5 km) was within 0.25% of the expected (randomized) fire locations for all post‐fire years, except year 3 when average DA was 0.64% (SE, 0.36) higher in the observed dataset (Figure 3C). The second nearest buffer area (>0.5–1.0 km) was ~0.6% higher than the expected in years 3 and 4. Comparing DA with distance from fire by year, pairwise tests indicated that the observed DA (in percentage) was not significantly greater in the two buffer areas nearest the fire compared to areas further away (*p* > 0.05 after “Holm–Bonferroni” correction). Examining the variability among fires in the buffer area nearest the fire (0–0.5 km), seven fires (of 61) had DA 2% higher than expected and one fire had DA 5.0% higher than expected from 3 to 5 years post‐fire. Pairwise tests were similar for buffer widths of 1 km (i.e., not statistically different), but in the nearest buffer area (0–1.0 km), 14 fires had DA 2% higher than expected and four fires had DA 5% higher than expected (Appendix S1: Figure S9C).

### DFB population pressure from burned and unburned sources with distance from fire

**FIGURE 4:**
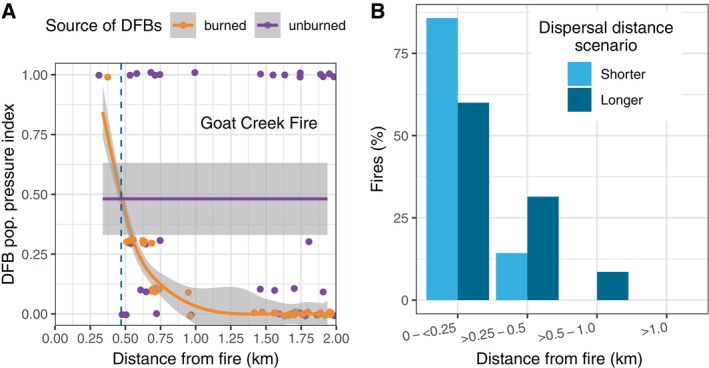
(A) Douglas‐fir beetle (DFB) population pressure from burned and unburned sources on areas (dots) of Douglas‐fir forest infested by DFB (i.e., aerial survey polygons) with distance from the fire for an example fire (Goat Creek), illustrating where the dominant source of DFB pressure switches from burned to unburned (~0.45 km) under a longer range DFB dispersal scenario (see Appendix S1: Figure S5 for a year‐by‐year example). Lines are generalized additive models, and gray shading is the 95% CI. (B) Percent of fires by distance from fire at which DFB pressure switched from burned to unburned sources under shorter and longer range DFB dispersal scenarios (see *Methods*).

For individual patches of DF mortality in unburned areas (i.e., IDS polygons), DFB pressure <0.5 km from fire was from a mix of unburned and burned sources, whereas ≥0.5 km from fire, DFBs were overwhelmingly from unburned sources (Appendix S1: Figure S10). After accounting for DFB dispersal to unburned areas, DFB pressure from burned areas decreased precipitously with distance from fire (see Figure 4A). The dominant source of DFB pressure transitioned from burned to unburned sources <0.25 km from fire for 86% of fires under the shorter range dispersal scenario and 60% of fires under the longer range dispersal scenario during the period 2–6 years post‐fire (Figure 4B). Greater than 1 km from fire, the dominant source of DFB pressure on new patches of DF mortality was from unburned sources under both dispersal scenarios (Figure 4B; Appendix S1: Figure S10).

### Identifying individual fires with DFB spillover

**FIGURE 5:**
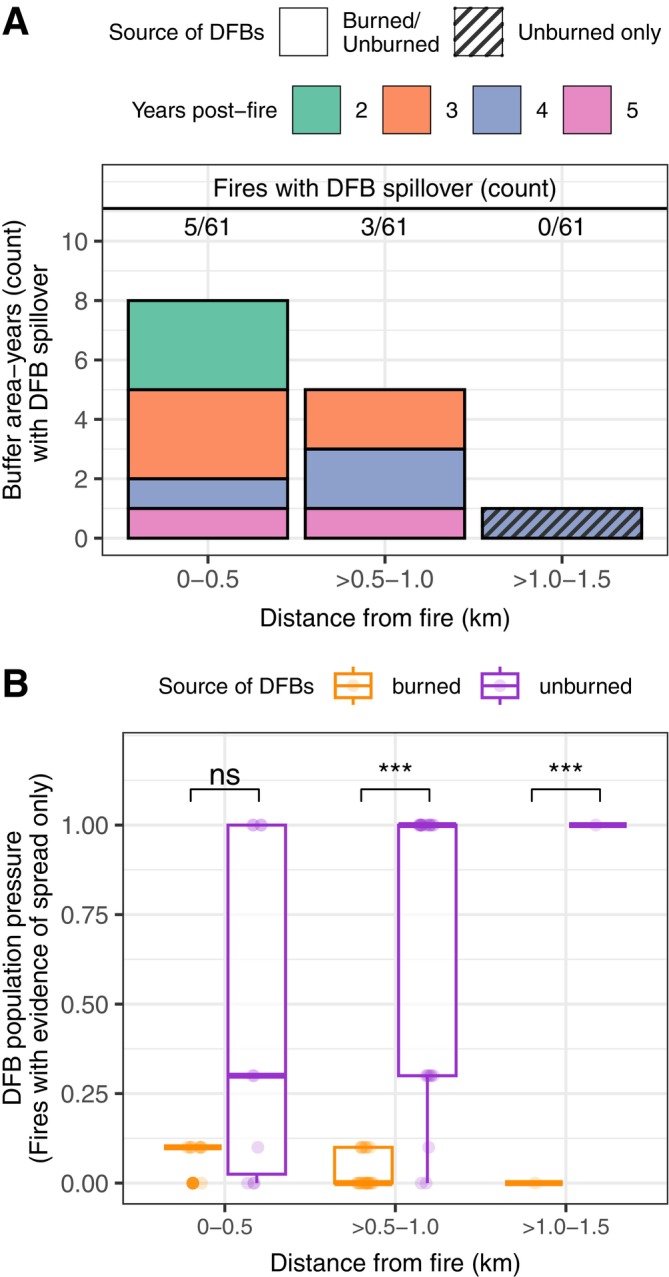
(A) Count of buffer area‐years (*y*‐axis) that met our criteria for Douglas‐fir beetle (DFB) spillover, sources of DFBs (burned and/or unburned; see panel B), and the count of unique fires with DFB spillover with time since fire and distance from fire (colors) based on the spatiotemporal progression of Douglas‐fir (DF) damage area from DFB. Individual fires may have multiple buffer‐years with DFB spillover. The analysis extracted DF damage area in 0.5‐km width buffer areas from 0 to 10 km from the fire perimeter by year (i.e., buffer area‐years; see Appendix S1: Figure S12 for 1.0‐km width buffer areas). (B) DFB population pressure from burned and unburned sources on individual aerial insect survey polygons for fires with evidence for DFB spillover only (see Appendix S1: Figure S11 for individual fires), and the results from pairwise tests of DFB source within buffer areas from a linear mixed model (ns, not significant; ***p < 0.001). In the boxplots, the thick horizontal line is the median, the box represents the interquartile range (IQR, 25th–75th percentiles) of the distribution, the whiskers extend no further than ±1.5 times the IQR, and the dots are outliers.

Our criteria for DFB spillover were met for 8% (5/61) and 5% (3/61) of fires in the areas 0–0.5 km and 0.5–1 km from fire, respectively, with spillover possible from 2 to 5 years post‐fire (Figure 5A). When examining 1 km buffer areas, 15% (9/61) of fires met our criteria for spillover to the area 0–1 km from fire (Appendix S1: Figure S12). For fires with DFB spillover, DFB pressure was mixed (unburned and burned sources) < 1 km from the fire, whereas >1 km from fire, DFB pressure from unburned sources was significantly greater than burned sources (*p* < 0.001; Figure 5B; see 1‐km buffer areas in Appendix S1: Figure S12). No fires met our criteria for spillover >1 km from fire due to closer proximity of DFB from unburned sources (Figure 5). The sum of DF DA in buffer areas meeting our criteria for DFB spillover, including DFBs from burned and unburned sources, was 280 ha when analyzed with 0.5‐km width buffer areas and 378 ha when analyzed with 1‐km buffer areas, representing 0.2% (0.5‐km buffers) and 0.3% (1‐km buffers) of total DF DA from DFB in the study area from 2002 to 2022.

### Drivers of DFB spillover

Among the many biophysical variables expected to influence DFB spillover (Appendix S1: Table S5), DF DA in the prior year in the same buffer area was the only variable that effected DFB spillover and it had a strong, positive influence on the likelihood for detection of DFB spillover, as indicated by the GLMM (1‐km width buffer areas only; β = 1.333, *p* < 0.01; Appendix S1: Table S6). All variables describing host availability and climate effects on host stress and DFB survival were not statistically significant (*p* > 0.136).

### Site disturbance agents confounding attribution of DFB spillover

**FIGURE 6:**
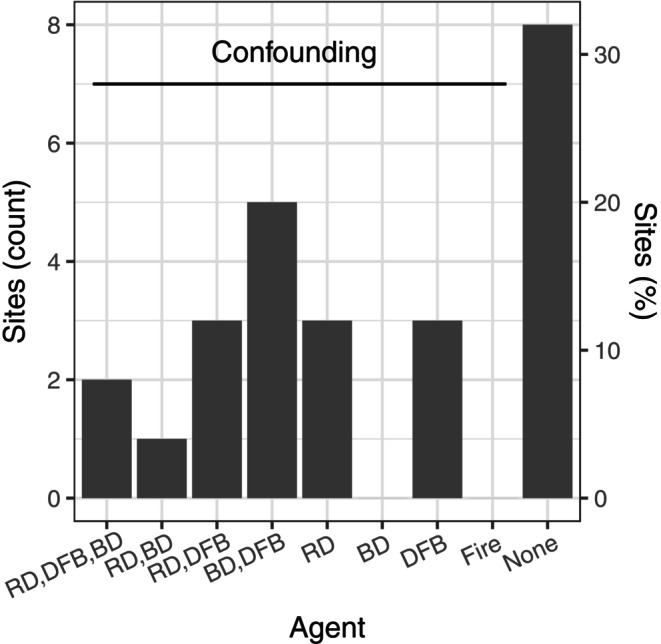
Count and percent of field plots with agents potentially confounding the attribution of Douglas‐fir (DF) mortality to spillover of Douglas‐fir beetles (DFB) from fire‐injured trees. Douglas‐fir trees affected by root disease (RD), DF trees blowdown (BD) after the fire year (2016 or 2017), and DFB infestation <5 years prior to the fire may have supported localized populations of DFB in the stand prior to or immediately after fire. Fire was included to account for undetected small fires or spot fires excluded from the fire perimeter polygon. The “none” category indicates that no potential confounding factors were identifed at the site, and DFB is assumed to have arrived from a neighboring stand, either from burned areas inside or from unburned areas outside of the fire perimeter.

Of the patches of DF tree mortality that we assessed in the field outside of fire perimeters, 68% (17 of 25) had prior disturbance agents potentially confounding the attribution of DFB activity to spread from burned areas (or other locations with DFB activity; Figure 6). Prior disturbance agents included root disease, blown down trees (infested by DFB and similar decay class as standing dead trees with DFB infestation in the last 5 years), and DFB activity in the 5 years prior to fire in IDS data (confirmed by field assessment of decay class). We found no evidence in the remaining 32% (8 of 25) of sites for disturbance agents in the prior c. 10 years (Figure 6).

## DISCUSSION

Our spatiotemporal analysis of DF tree mortality surrounding fires in the NRM did not indicate that wildfire initiated broad‐scale outbreaks of DFB that spilled over to unburned areas. Instead, we found that DFB spillover from wildfires was uncommon (i.e., generally a neutral interaction), but spillover may be associated with limited amounts of spatially diffuse DF tree mortality over relatively short distances from burned areas (<1.0 km) following some fires (Figures 2, 3, 4, 5). Additionally, the amount of DF mortality immediately surrounding (<1.0 km) the 61 study fires with and without evidence of spillover was relatively insignificant (2%) compared to the amount of mortality in the NRM during the study period. By evaluating the frequency of occurrence and potential distance of DFB spillover into unburned areas surrounding wildfires, our results provide ecological insights into interactions between fire and bark beetles that have received insufficient study but have important implications for pre‐fire planning and post‐fire forest management.

If epidemic‐level populations of DFB were dispersing from burned to unburned areas, we expected high levels of DF tree mortality immediately surrounding wildfires (<1.0 km) compared to further away due to the energy required for dispersal, the greater mortality rates of longer range dispersal, and abundant suitable DF forest for DFB surrounding our study fires (Jones et al., 2019). Across all fires we did not detect elevated levels of DF mortality immediately surrounding wildfires relative to further away in the spillover analysis (Figure 3), suggesting that the peak in DF mortality 3–4 years post‐fire in the regional (Figure 2) and spillover analysis (Figure 3) was poorly associated with fire activity. Prior studies of post‐fire bark beetle population dynamics in fire‐injured trees inform our findings. In ponderosa pine and lodgepole pine forests, attack and reproductive success of their respective bark beetle species responded positively to the pulse of fire‐injured trees, but populations quickly declined within a few years when suitable breeding material and interspecific competition restricted growth of bark beetle populations (Davis et al., 2012; Lerch et al., 2016; Powell et al., 2012). The reproductive and attack success of DFB in post‐fire environments has not been studied, but DFB population growth is similarly constrained by the availability of stressed trees with low resistance to attack and interspecific competition in available breeding material (Aukema et al., 2016). Successful DFB attacks on healthy DF trees are limited to periods of epidemic‐level DFB populations (Furniss et al., 1979), and we found minimal evidence for epidemic‐level population spillover to unburned areas in the spatiotemporal pattern of tree mortality. Though DFBs contribute to post‐fire tree mortality (Busby et al., 2024; Hood & Bentz, 2007), DFB population growth within fire‐injured trees is likely short‐lived (~2 years) in response to the “resource pulse” (Davis et al., 2012). In summary, DFB populations in burned areas are likely insufficient to spillover and initiate widespread, mass‐attacks that result in mortality of large quantities of healthy DF trees in unburned areas surrounding fires, even during a period of amplified wildfire activity and drought.

Our finding that DF tree mortality from DFB increased both within and beyond the common flight distance of DFB following fire (Figures 2 and 3) is supporting evidence for the local reproduction hypothesis (Table 1). Synchronous annual to multi‐year increases in DF tree mortality at multiple distances from fire is consistent with a broader scale process initiating DFB activity, such as warmer and drier climate conditions. In our study, we did not undertake a rigorous analysis of climate effects on DFB and fire activity, but we note that fires occurred in warm and drier years (Appendix S1: Figure S8), a result consistent with research examining climate effects on fire activity in dry forests in the NRM (Morgan et al., 2008). Similarly, elevated DFB activity 3 years after fire was qualitatively associated with warmer and drier conditions in the preceding years and year that DFB activity peaked at multiple distances from fire (Appendix S1: Figure S8). In agreement, DFB outbreaks (as inferred from higher levels of DF mortality) were more likely following warm and dry spring and summer conditions during the year of the DFB outbreak and two prior years in the western United States (Howe et al., 2024). In further support for the local reproduction hypothesis, we found that DF DA immediately surrounding fire (<0.5 km) was actually lower than areas further away (>0.5–5 km) from 2 to 6 years post‐fire, potentially suggesting that DFB may be dispersing from unburned areas near fire into burned areas. Though we found evidence supporting the local reproduction hypothesis, the hypotheses are not mutually exclusive. Our finding that DF mortality was slightly elevated 1–2 km from fire (years 3 and 4 only) but not significantly different in the spillover analysis may indicate DFB dispersal from fire over multiple years or longer distance dispersal events, a finding that may support the spillover hypothesis. Importantly, the amount of mortality in these areas was still consistent with the relatively low levels of spatially diffuse DF mortality observed in our study, and the area 1–2 km from burned areas was more strongly influenced by more proximate sources of DFB from unburned areas (Figure 4). Though the results from the regional and spillover analyses provide stronger support for the local reproduction hypothesis, we did find supporting evidence for the spillover hypothesis following individual fires.

A minority of individual fires (8%–15% depending on buffer width) selected for analysis met our criteria for DFB spillover and all spillover buffer‐areas occurred within 1.0 km of all fires (Figure 5). Importantly, the percentage of fires with DFB spillover was estimated based on a subset of fires in the NRM with a relatively high amount of susceptible DF forest surrounding each fire and presumably a higher likelihood for DFB spillover, which collectively indicates that our percent estimate of fires with spillover is likely higher than expected if we included all fires in DF forests in the NRM. For our subset of study fires with spillover, spillover resulted in relatively small amounts of DF tree mortality (≤0.3% of mortality in the study area during the study period). Consistent with the period of DFB reproduction and depletion of fire‐injured host trees (Hood & Bentz, 2007), DFB spillover was detected in years 2–5 post‐fire (Figure 5). The primary evidence supporting spillover in individual fires was high DF DA (>90th percentile) immediately surrounding individual fires relative to areas further away (>1–10 km; Figure 5A). Within spillover areas, DFB pressure was from burned and unburned sources <1.0 km from fire but dominated by unburned sources >0.5 km from fire due to the closer proximity of DFBs from unburned sources with distance from fire (Figure 5B). Within areas where spillover may occur, we found field evidence in most sites (68%) for disturbances agents that may support localized populations of DFBs, potentially confounding but not eliminating the attribution of DF tree mortality to DFB spillover. For example, we found patches of forest infested by root diseases that likely sustained small DFB populations pre‐fire and represent an unburned source of DFB following fire (Schmitz & Gibson, 1996). Due to the complexity of DFB population dynamics and dispersal, longitudinal field surveys of DFB reproductive success and DF mortality from DFB in burned and adjacent unburned areas are needed to better understand the spatiotemporal dynamics of post‐fire DFB activity in the areas immediately surround fires, such as the area <0.5 km from fire but up to 1.0 km.

We did not find evidence that DFB spillover was contingent on physiological stress of host trees from drought or other causes as expected in the stress‐spillover hypothesis (see *Results* for GLMM in Appendix S1: Table S6). Douglas‐fir tree host stress is one of the most important factors determining resistance to DFB (Schmitz & Gibson, 1996). As such, we expected that severity of drought conditions following fire, as has been recently observed in the NRM (Williams et al., 2022), may condition DF trees to be more susceptible to DFB spillover. Instead, we only found that DFB spillover was positively associated with DFB activity in the prior year in the same area (1 km width buffer areas only; Appendix S1: Table S6). When spillover was detected in the second year after fire, DFB activity in the prior year (i.e., one year fire) in the same area was most likely from DFBs from unburned sources, because DFBs were colonizing fire‐injured trees in the first year after fire, potentially reducing the number of detected spillover events. No evidence in support of the stress‐spillover hypothesis may be explained by similar climate drivers of burned area and DFB activity, missing evidence or criteria for detection of spillover, false detection of spillover, or elevated host tree stress due to post‐2000 shifts toward warmer and drier conditions in the region. In the context of climate change, unfavorable annual to decadal climate conditions that increase DF host stress and reduce tree vigor, especially in marginal sites (e.g., edges of the species distribution; Bennett et al., 2023), in combination with ever‐present DFB populations in unburned forests may continue to challenge the detection of biophysical factors influencing spillover.

Our criteria for detection of spillover were designed to detect high levels of DF mortality radiating away from fire in a linear progression. Unknown quantities of DFBs do disperse at irregular distances (long‐range dispersal), suggesting that DFBs from burned sources may mix with DFBs from unburned sources in areas within and beyond their common flight distance, potentially resulting in diffuse tree mortality. In addition, non‐linear spread of DFB from fires over multiple post‐fire years may occur but is difficult to track directly with the remote sensing data sources used our study. Such factors may partially explain the elevated levels of DF mortality in areas 1–2 km from fire in the post‐fire years 3–4 (Figure 3). Given the difficulty of tracking the source of DF tree mortality several years post‐fire, our buffer analysis may have underestimated the effects of mild, diffuse spillover. However, we expect long‐range dispersal to be a relatively minor contributor to DF tree mortality in unburned areas due to the fitness requirements and abundant suitable habitat surrounding burned areas.

### Management implications

Managing bark beetle populations following and surrounding fires is a growing concern for land managers. For example, the Ministry of Forests in British Columbia, Canada, issued a statement that DFB population growth in susceptible, fire‐injured host trees will spread beyond fire perimeters and colonize live and seemingly healthy trees (BC Ministry of Forests, 2023). Managers need additional information to evaluate whether mitigation actions aimed at reducing post‐fire risk of DFB spillover are needed to protect high‐value DF forests adjacent to wildfire perimeters.

The unburned areas immediately surrounding fires are likely influenced by DFBs emigrating from burned and unburned sources (Powell et al., 2012), and our study directly addresses the potential for DFB populations in fire‐injured trees to effect unburned forests. Using a shorter and longer range dispersal scenario of DFBs from burned and unburned sources, we estimated that the dominant source of DFBs transitioned from burned to unburned sources at <0.25 km for most fires and <0.5 km for nearly all fires (Figure 4), with limited influence of burned sources >0.5 km. For post‐fire management applications, our distance estimates need to be considered in the context of the spatial accuracy of IDS polygons (e.g., 79% accuracy within 0.5 km; Johnson & Ross, 2008) and the spatial resolution of the DFB population pressure weights matrix (240‐m raster; see *Methods* for details), potentially suggesting that the spatial accuracy of our estimates is within ±0.25 km (not based on formal analysis). Despite uncertainties associated with the spatial datasets, the agreement between the shorter and longer range dispersal scenarios suggests that DFBs from burned sources have the strongest influence on areas <0.5 km from burned areas. Though most individual fires did not meet our criteria for spillover, we did identify 8%–15% (range from sensitivity analysis) of fires a higher likelihood for spillover. To protect high‐risk or high‐value resources (e.g., trees near homes, with cultural value, or in ski areas), land managers may consider monitoring DFB populations in fire‐injured trees and assessing the susceptibility of DF forests surrounding individual fires to determine the risk for DFB emigration from burned area to unburned forests.

For protecting DF forests from DFB, current available management practices focus on protecting high‐value trees or forest stands over the shorter term (years to decades), with limited capacity to reduce resistance (tree mortality) to bark beetles across large, forested landscapes over the longer term (multiple decades to century). For site‐specific areas where resource value is high and tolerance to risk is low, mitigation treatments, such as anti‐aggregation pheromones (e.g., MCH), sanitation harvest of infested trees, and pheromone‐baited funnel traps, may help protect high‐value DF trees and stands near fires (<0.5 km) in the five years after fire (Fettig et al., 2022). Shifting forest management practices or facilitating the return of other disturbance processes, such as active fire regimes, that create conditions less susceptible to DFB, such as greater species and structural diversity, may promote longer term resilience across the forest landscape as future warmer and drier climate conditions will inevitably include periods of high DFB activity (Bentz et al., 2010; Raffa et al., 2008; Windmuller‐Campione et al., 2021).

## CONCLUSION

Tree mortality from bark beetle and fire activity have both increased rapidly since 2000 and overlapped closely in space and time, with much speculation about outcomes of disturbance interactions. Our study is one of very few that evaluate the potential for bark beetles to spillover into unburned, green forests. To address land managers concerns, we developed a multiple‐scale analytical framework that could be applied to insect spillover following fire or other disturbances (e.g., windstorms) where spatial data are available. Across many fire events, the spatiotemporal pattern of tree mortality did not indicate obvious effects of spillover‐caused mortality, but we did find individual fire events with short‐range spillover. Our results help land managers constrain the area and timescales of greatest risk for DFB spillover. Given the complexity of tracking bark beetle populations post‐fire at fine spatial scales, complementary data sources are needed to improve our understanding of spatiotemporal dynamics of bark beetles and geographic variability in fire–beetle relationships.

## AUTHOR CONTRIBUTIONS

Robert A. Andrus, Joel Egan, Brytten Steed, Laura Lowrey, Cameron E. Naficy, and Arjan Meddens designed the study. Robert A. Andrus and Nathan Ivy collected the field data. Robert A. Andrus performed the statistical analysis and wrote the initial draft of the manuscript. All authors contributed to writing and revising the manuscript.

## CONFLICT OF INTEREST STATEMENT

The authors declare no conflicts of interest.

## Supporting information


Appendix S1.

## Data Availability

Our study collected one field dataset (Andrus, 2025), available in Ag Data Commons at https://doi.org/10.15482/USDA.ADC/29075840.v1. Additionally, our study used publicly available datasets as described in Appendix S1: Table S2, and details for how we queried these data are in *Methods* *:* *Data and data processing*.
