# Supplementary material for: Limited tree mortality in unburned areas linked to bark beetle spillover from wildfires
Source: Ecol Appl. 2025 Jul 2;35(5):e70066. doi: 10.1002/eap.70066 (PMC12223477; doi:10.1002/eap.70066)
Supplement: Supplementary file 1 — Appendix S1. [file EAP-35-e70066-s001.pdf]

APPENDIX S1

Authors: Robert A. Andrus, Joel Egan, Nathan Ivy, Laura Lowrey, Cameron E. Naficy, Brytten Steed, Arjan Meddens

Title: Limited tree mortality in unburned areas linked to bark beetle spillover from wildfires

Journal: Ecological Applications

Table S1: Proportion of Douglas-fir (DF) trees likely killed (mass-attacked) by Douglas-fir beetle (DFB) after fire-injury from studies in the Northern Rocky Mountains, USA. Estimates are from two or five years after fire, and fire-injured DF mortality from DFB typically peaks two years after fire (Hood and Bentz 2007).

Fire (year)	State	DF tree mortality attributed to DFB after fire injury (%)	Citation
Lowman (1989)	ID	15% (29 of 188)	(Weatherby et al. 1994)
Yellowstone (1988)	WY	76% (73 of 96)	(Ryan and Amman 1996)
Mussigbrod (2000)	MT	19.6%^	(Hood and Bentz 2007)
Mussigbrod (2000)	MT	NA*	(Bulaon 2003)
Blodgett Trailhead (2000)	MT	NA*	(Bulaon 2003)
Crooked (2000)	ID	NA*	(Bulaon 2003)
Elizabeth (2000)	ID	NA*	(Bulaon 2003)
Cave Gulch (2000)	MT	NA*	(Bulaon 2003)
Maudlow-Toston (2000)	MT	NA*	(Bulaon 2003)
Cliff Point (2000)	MT	NA*	(Bulaon 2003)
Lydia Mountain (2000)	MT	NA*	(Bulaon 2003)
Stone Hill (2000)	MT	NA*	(Bulaon 2003)
Flat Creek (2000)	MT	NA*	(Bulaon 2003)
Landowner Mountain (2000)	MT	NA*	(Bulaon 2003)
Ninemile (2000)	MT	NA*	(Bulaon 2003)
Burnt Flats (2000)	ID	NA*	(Bulaon 2003)
Moose (2001)	MT	43%^	(Hood and Bentz 2007)
Green Knoll (2001)	MT	73%^	(Hood and Bentz 2007)
Castle Rock (2007)	ID	37% (57 of 154)	(Lazarus 2011)
Halstead (2012)	ID	16%	(Lowrey et al. 2015)

* 10 out of 13 fires had 30-50% of fire-injured DF trees attacked by DFB one to two years after fire, with generally greater mortality in year two.
^ of suitable trees (i.e., fire-injured, live and > 23 cm diameter at breast height)

Table S2: Characteristics of the spatial datasets used in the spatial analysis.

Dataset (source)	Description	Attributes	Data type	Years (frequency)
Insect and disease survey (FHP 2023)[1]	Biotic damage from pests and pathogens in forest areas, and spatial extent of area surveyed	Damage area for Douglas-fir beetle (11007) and area surveyed	Polygon	1998-2022 (annual)
Fire perimeters (MTBS 2023)[2]	Burned Areas Boundaries Dataset	Area burned	Polygon	1993-2017 (annual)
Individual tree species parameter maps (Krist et al. 2014)[3]	Stand structure and composition metrics	Basal area (m^2 ha^{-1}) of Douglas-fir; Percent of total basal area in Douglas-fir; Stand density index (SDI)	Raster (240 X 240 m)	c. 2002
Forest cover (Hansen et al. 2013) [4]	Percent cover of forest. Data aggregated from 30 m to 240 m	Percent cover of forest	Raster (30 X 30 m)	c. 2000
Timber harvest (USDA 2022a), Hazardous Fuel Treatments (USDA 2022b), Silviculture Timber Stand Improvement (USDA 2022c) [5]	Forest management activities	All forest management activities that remove tree biomass (e.g., clear-cut, selection cut)	Polygon	1990-2022
SWE (Broxton et al. 2019) [6]	Gridded snow-water equivalent (SWE)	April 1 SWE	Raster (4 X 4 km)	1982-2021
PRISM (PRISM 2023) [7]	Monthly climate variables	Minimum and mean temperature and precipitation	Raster (4 X 4 km)	2000-2022
Ecoregions (Level III (EPA 2010) [8]	Boundaries of four ecoregions in the study area	Ecoregion name	Polygon	2006
USA Federal Lands (ESRI 2024) [9]	All lands managed by the USA Federal Government	Federal Lands	Polygon	2024

[1] https://www.fs.usda.gov/science-technology/data-tools-products/fhp-mapping-reporting/detection-surveys
[2] https://www.mtbs.gov/direct-download
[3] https://www.fs.usda.gov/science-technology/data-tools-products/fhp-mapping-reporting/individual-tree-species-parameter-maps
[4] https://data.globalforestwatch.org/documents/941f17325a494ed78c4817f9bb20f33a/explore
[5] http://data.fs.usda.gov/geodata/edw/datasets.php
[6] https://nsidc.org/data/nsidc-0719/versions/1
[7] http://prism.oregonstate.edu
[8] https://www.epa.gov/eco-research/level-iii-and-iv-ecoregions-continental-united-states
[9] https://atlas.eia.gov/datasets/5e92f2e0930848faa40480bcb4fdc44e/explore

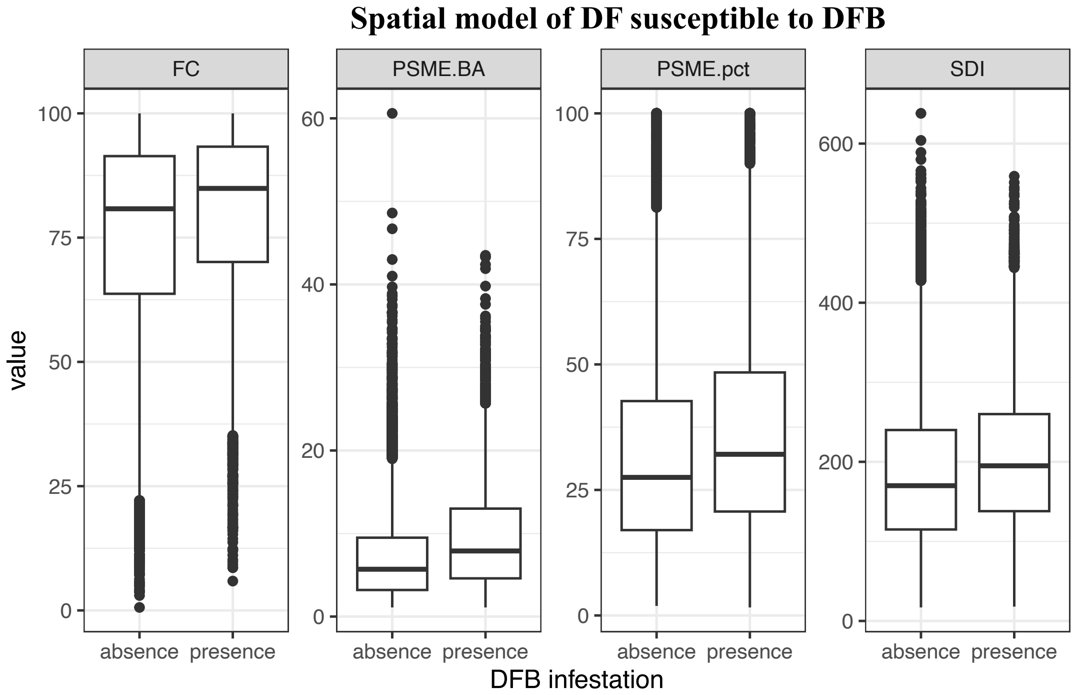

Fig. S1: Presence/absence of DFB infestation for four stand composition and structure variables, including forest cover (%), basal area of Douglas fir (PSME.BA, m^2/ha), percent of total stand basal area in Douglas-fir (PSME.pct), and all species stand density index (SDI).

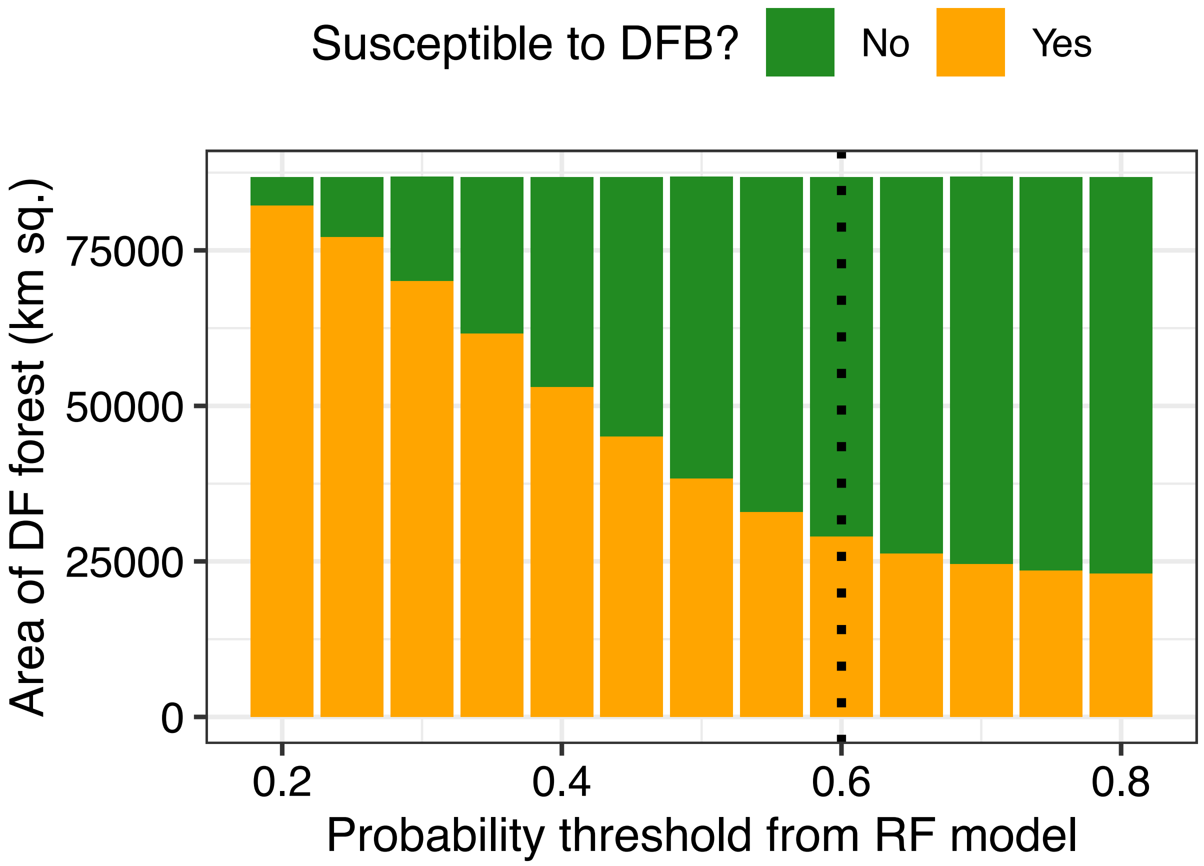

Fig. S2: Sensitivity analysis of Douglas-fir (DF) forest area susceptible (orange) or not susceptible (green) to Douglas-fir beetle (DFB) for multiple probability thresholds from the random forest (RF) model (bars). We defined susceptible DF forests as a probability threshold >= 0.6 (vertical dashed line).

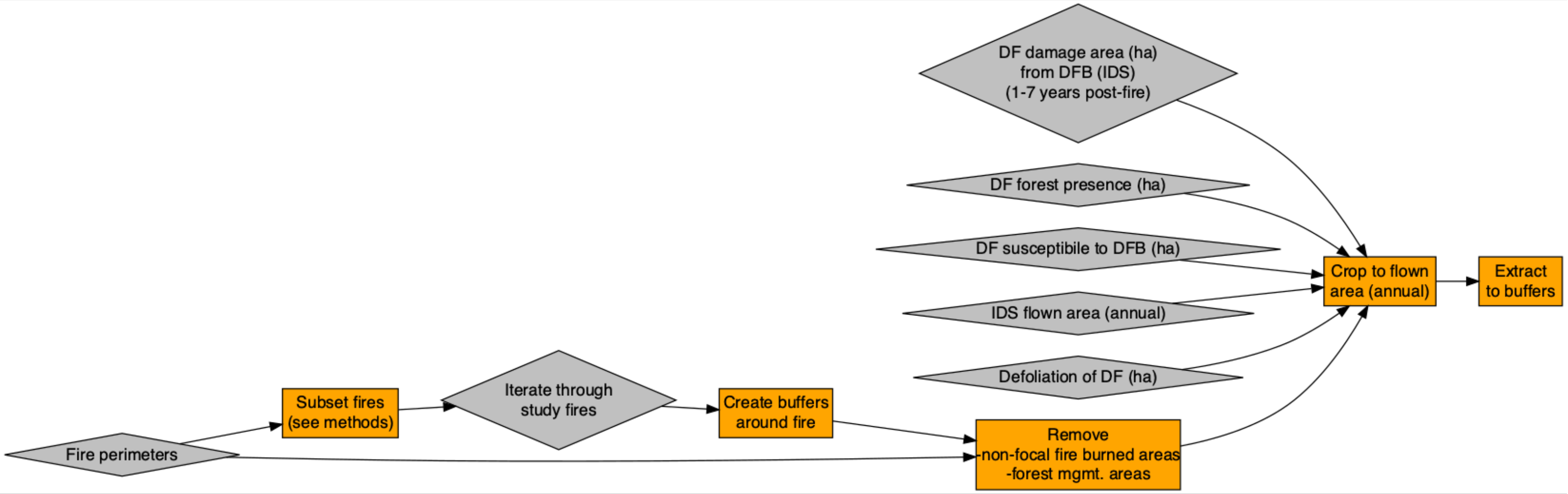

Fig. S3: Flow diagram illustrating input datasets (gray diamonds) and functions (orange rectangles) used in the spatiotemporal analysis of Douglas-fir (DF) damage area from Douglas-fir beetle (DFB). In the final step ('Extract to buffer'), the following were extracted from within each buffer area for each year one to six years post-fire: fire attributes (fire ID, fire name, total area), Douglas-fir (DF) damage area (ha) from Douglas-fir beetle (DFB) by year, area of DF forest presence (> 1 m^2/ha), area of DF forest susceptible to DFB (from Random Forest model), area flown by the insect and disease survey (IDS), and area of DF forest defoliated by western spruce budworm and Douglas-fir Tussock Moth. Buffer areas were 1 km increments from 0 to 10 km from fire.

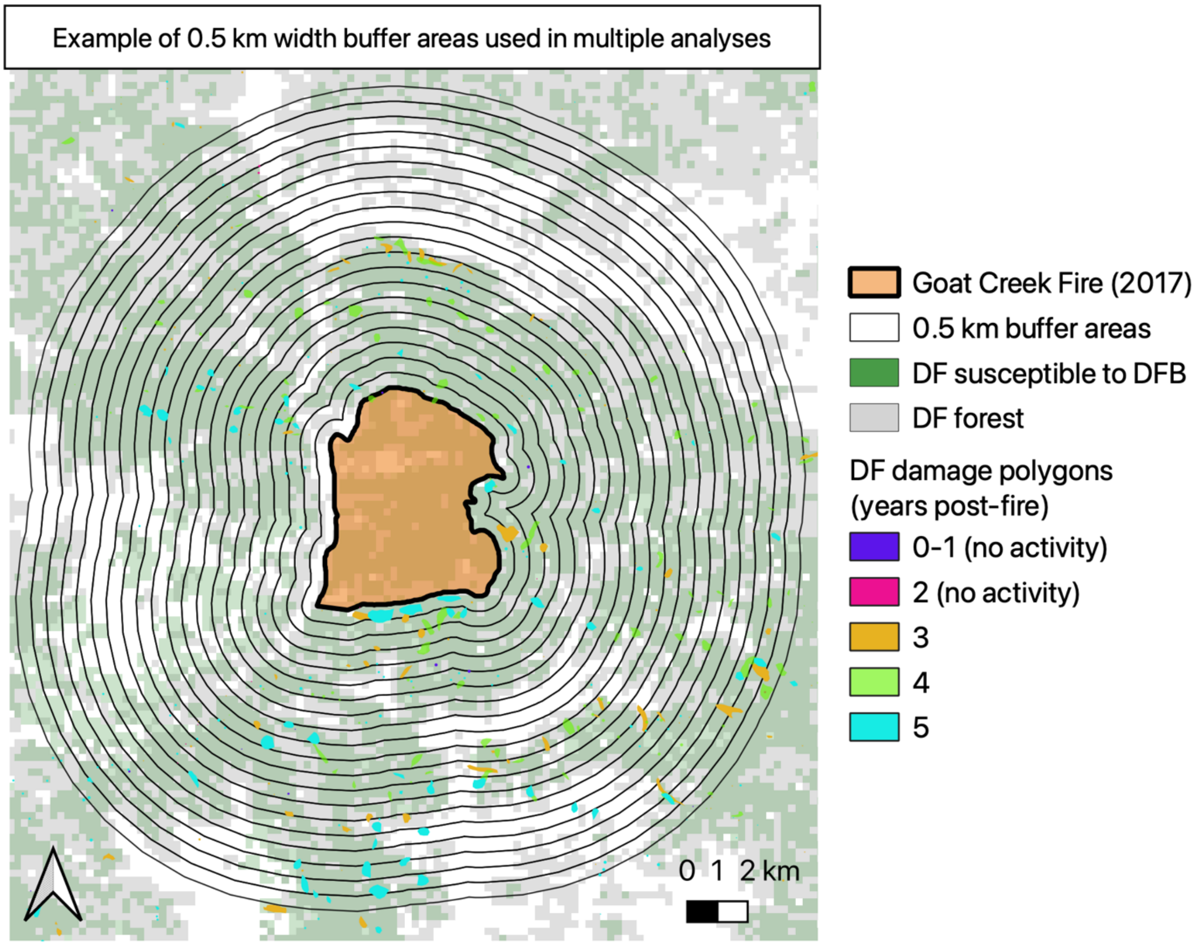

Fig. S4: (A) Example of 0.5 km width buffer areas used to extract area of Douglas-fir (DF) forest susceptible to Douglas-fir beetle (DFB), DF forest, and polygons of DF damage area from DFB by year post-fire. DF damage area is from the USDA Insect and Disease Surveys (FHP 2023). An area 10 to 15 km from the fire perimeter was also included, but is not visible on this map.

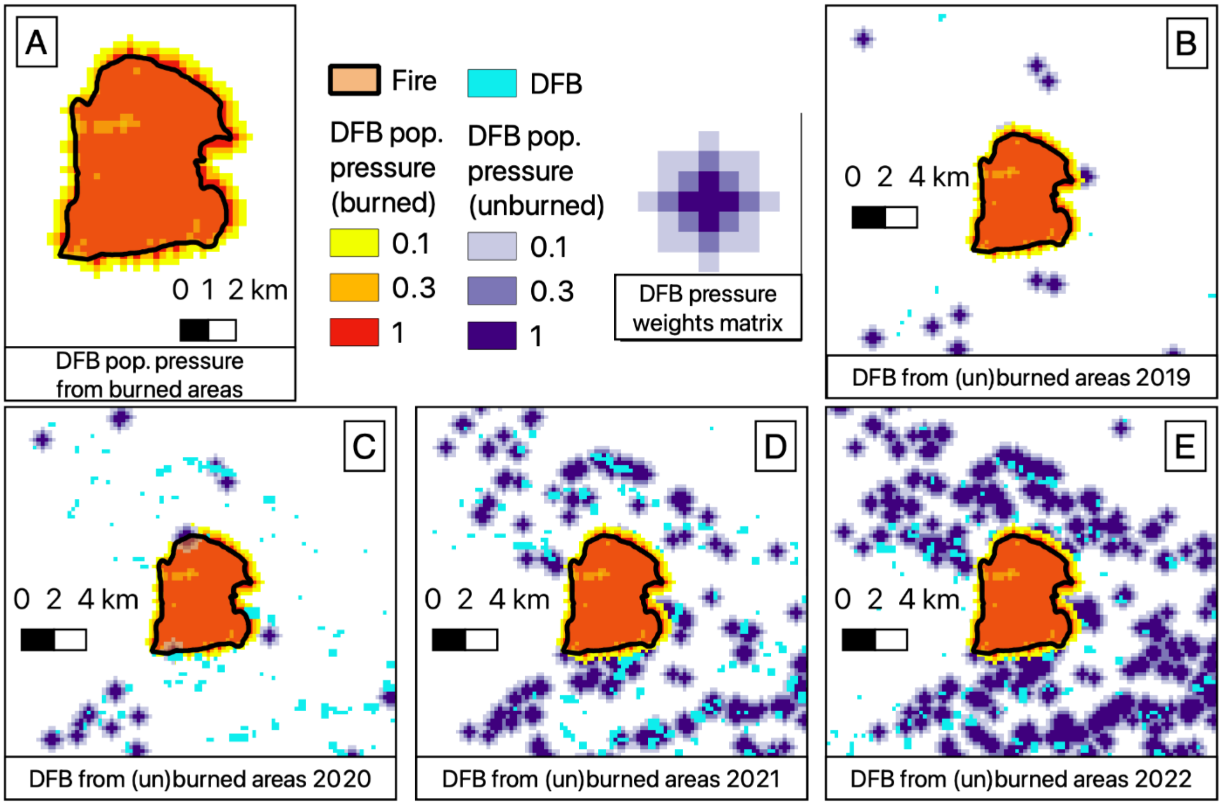

Fig. S5: Douglas-fire beetle population pressure from **(A)** burned areas only and **(B-E)** burned and unburned areas on current year DFB activity (cyan) for each year 2019 to 2022 (see panel caption) in the Goat Creek Fire (2017). Panels **(B-E)** illustrate that DFB population pressure originating from unburned areas increases simultaneously at distances near (e.g., 0-0.5 km) and far (e.g., 4.5-5 km) from the fire perimeter and that distances near fire may be affected by DFB population pressure from burned and unburned areas simultaneously. The DFB population pressure weights matrix in this example is the shorter range dispersal scenario and is shown in the legend (240 m resolution). The source of DFB population pressure from burned and unburned areas was estimated differently because insect and disease aerial survey polygons are inconsistently mapped within fire perimeter (e.g., general mapping begins three years post-fire). For DFBs originating from burned areas **(A)**, DFB population pressure was estimated by applying a distance weighted raster (240 m resolution to match other datasets) to the DF forest susceptible to DFB that burned (all severities) within each fire perimeter. For DFBs originating from unburned areas **(B-E)**, DFB population pressure was estimated by applying the same distance weighted raster to DFB activity (presence/absence) from the two prior years. To balance primarily short-range dispersal (< 200 m) and account for some longer-range dispersal, the following weights were assigned by distance: 1.0 for <= 240 m, 0.3 for >240 to <=480, and 0.1 for >480 to <=720. Our method likely overestimates DFB activity from burned areas for three reasons. (i) DFBs prefer low to moderate severity burned trees with moister phloem (Hood and Bentz 2007) and we included all burn severity classes. (ii) Only 15-76% of fire-injured DF trees are infested by DFB (Table S1) and we assumed that DFBs infested the entire 240 m pixel. (iii) DFB activity is likely higher three to five years post-fire, and we assumed constant DFB pressure from burned areas during the two to six-year post-fire period.

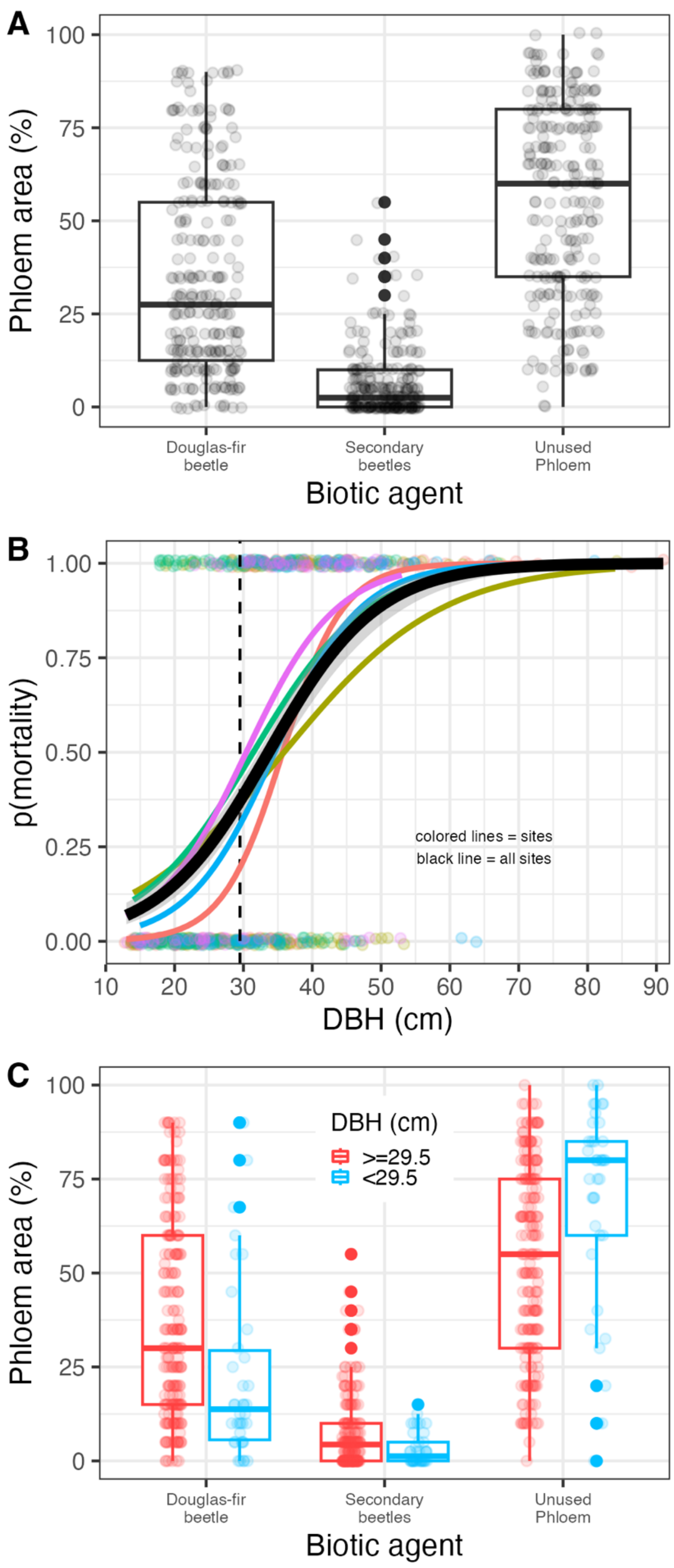

Fig. S6: (A) Percent of phloem area with reproductive galleries of Douglas-fir beetle (DFB), secondary beetles (primarily flatheaded woodborer and Scolytus spp.), and unused phloem (i.e., no galleries) from dead Douglas-fir (DF) trees, illustrating that DFB covered more phloem area than secondary beetles and was present on most dead trees. Dots are values from individual trees. **(B)** Probability of Douglas-fir tree infestation by Douglas-fir beetle (presence of DFB) by diameter at breast height (DBH) for five sample fires (colored lines) and overall (black line) with standard error (gray ribbon; logistic model; 647 total Douglas-fir trees). The vertical dashed line is the DBH probability threshold (29.5 cm) marking an increased likelihood for attack by DFB, which was identified by maximizing the true skill statistic (i.e., sensitivity + specificity - 1; 'pROC' package in R; (Allouche et al. 2006). All dead DF (290 trees) were killed within the last ~5 years. **(C)** Percent of phloem area with reproductive galleries of Douglas-fir beetle, secondary beetles (primarily flatheaded woodborer and Scolytus spp.), and unused phloem (i.e., no galleries) for dead DF trees greater than and less than 29.5 cm, illustrating that most main canopy dead DF trees were attacked by DFB. Dots are values from individual trees. In the boxplots, the thick horizontal line is the median, the box represents the interquartile range (25th–75th percentiles; IQR) of the distribution, the whiskers extend no further than +/-1.5 times the IQR, and the solid dots are outliers).

Table S3: Average and one standard deviation for stand structure and composition descriptors pre- and post-Douglas-fir beetle (DFB) in the 24 field plots (0.07 ha). Descriptors are for Douglas-fir (DF) only or all species. Quadratic mean diameter (QMD) is a measure of the average diameter in the stand. Stand density index (SDI; (Shaw 2000)) estimates the competition for resources (e.g., light and moisture) or stress that can increase the likelihood for DFB infestation (Negron 1999). In summary, prior to DFB infestations in the last c. five years, DF stands contained many susceptible DF trees (average, 734.0 DF stems ha^{-1} of trees > 29.5 cm), were composed primarily of DF (average, 87.2% DF basal area), and the main canopy cohort was of susceptible age (average, 129 years) (Table 2).

	**Pre-DFB**		**Post-DFB**	
**Stand variable**	**Average (SD)**	**Min.-Max.**	**Average (SD)**	**Min.-Max.**
DF QMD (cm)	21.5 (4.5)	13.9-31.3	17.6 (6.4)	6-26.4
DF stems ha^{-1}	1067.8 (444.8)	438.5-1853.3	445.9 (161.9)	155.6-735.6
All species stems ha^{-1}	1359.2 (619)	636.6-2645.5	689.2 (336.3)	282.9-1443
DF stems ha^{-1} > 29.5 cm DBH	734.0 (317.7)	212.2-1244.9	316.9 (209.4)	0-608.3
DF basal area (%)	87.2 (15.1)	53.2-100	71.2 (34.1)	3.1-100
DF basal area (m^2 ha^{-1})	35.2 (9.6)	22.7-59.5	12.4 (8.1)	0.4-31.1
DF basal area loss (%)	NA	NA	22.8 (9.3)	8.7-42.9
All species SDI	885.4 (229.4)	593.1-1396.7	405.2 (170.7)	82.1-680.2
Age susceptible DF (years)[τ]	NA	NA	129 (42.5)	68-250

[τ] Age of DF trees susceptible to DFB was estimated from one core collected at breast height (1.4 m) from two live DF trees that were representative of the largest cohort. Cores were processed following standard dendrological procedures and rings were counted to estimate tree age (Speer 2010).

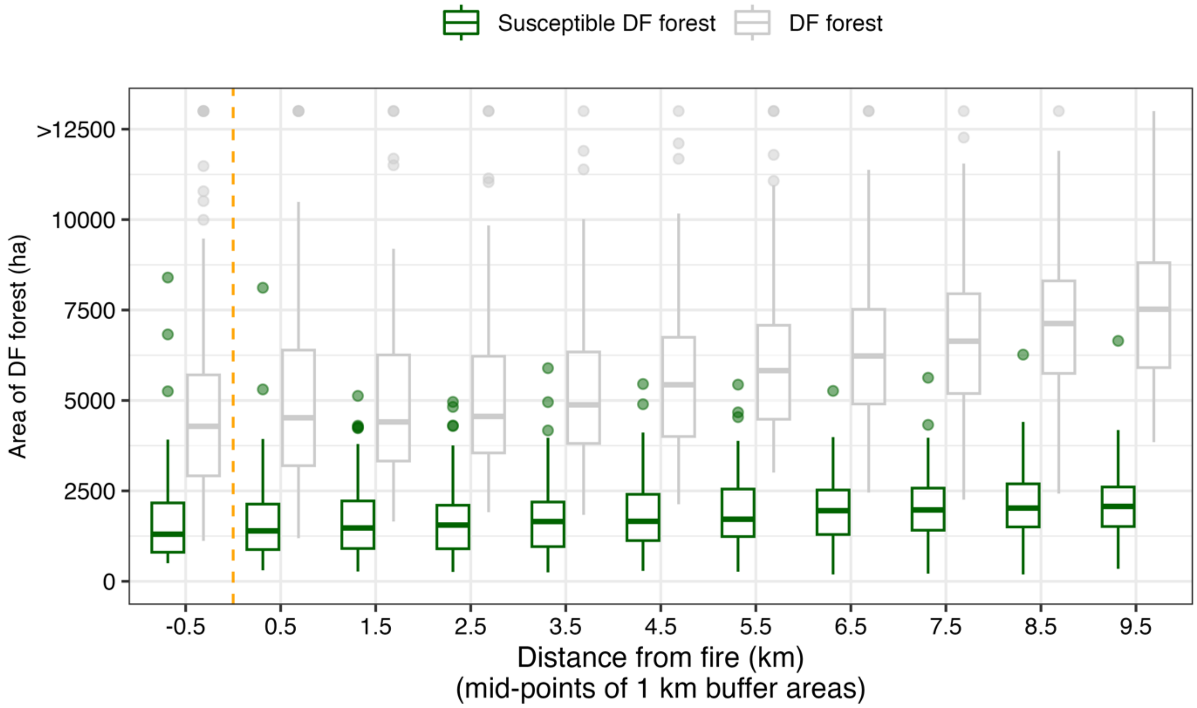

Fig. S7: Area (ha) of Douglas-fir (DF) forest and DF forest susceptible to Douglas-fir beetle (DFB) with distance from fire for the 62 study fires in the Northern Rocky Mountains, USA, illustrating that all study fires included DF forest susceptible to DFB. Values on x-axis are mid-points of 1 km buffer areas. The vertical dashed line (orange) indicate the fire perimeter (negative x-values are inside the fire perimeter, positive x-values are outside the fire perimeter).

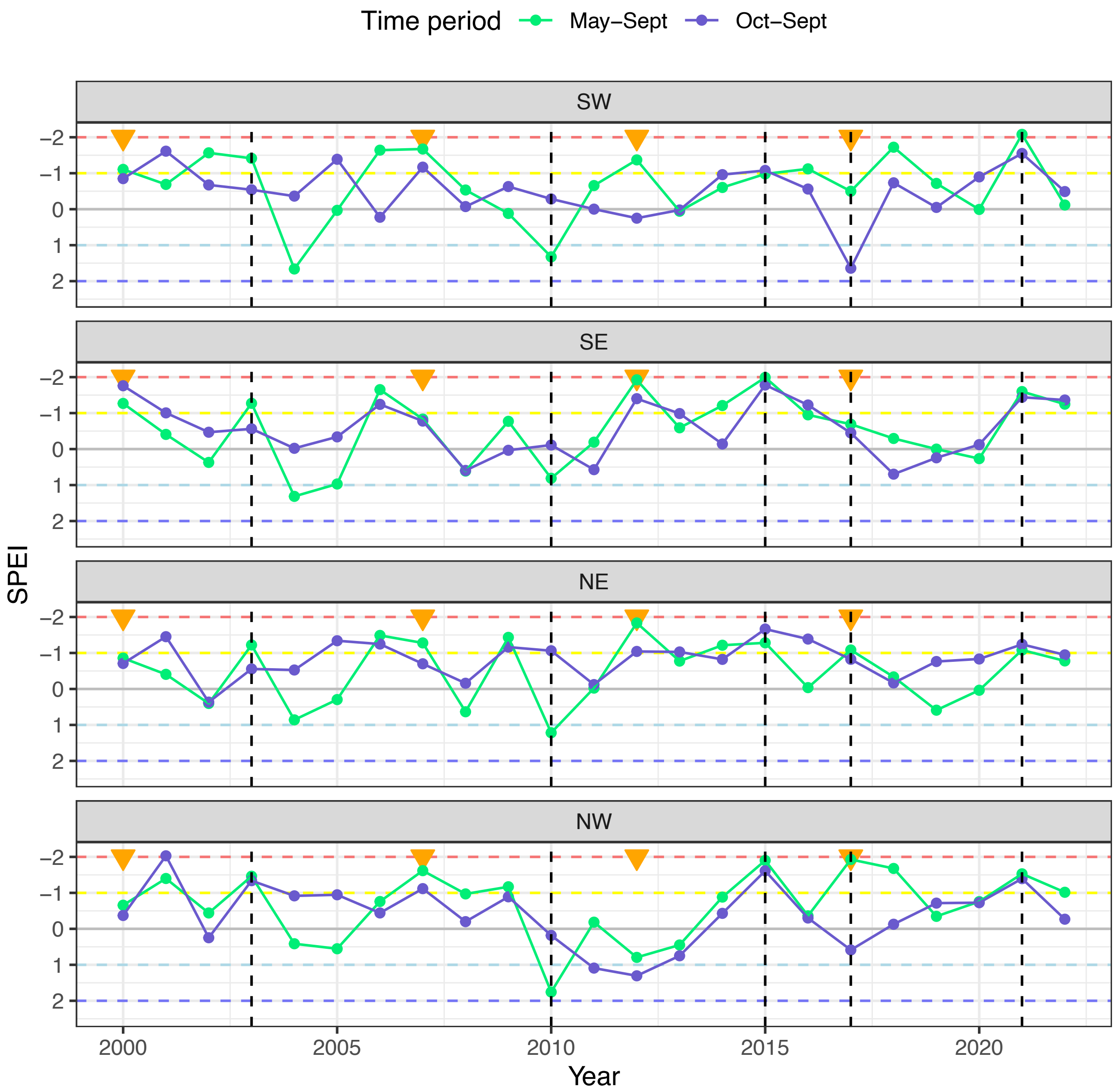

Fig. S8: Standardized Precipitation Evapotranspiration Index (SPEI), a drought index based on climatic data, from May to September (growing season) and October to September (water year) in the four ecoregions in the study area (Fig. 1). SPEI was computed from 1948 to 2022, and we display 2000 to 2022 to focus on the study period. Negative SPEI values indicate warmer and drier conditions, and positive values indicate cooler and wetter conditions. The y-axis was inverted. The inverted orange triangles indicate the four fire years with the most burned area (Fig. 2). Vertical lines indicate peaks in Douglas-fir mortality from Douglas-fir beetle near fire (<1.0 km or gray line in Fig. 2). Though peaks in Douglas-fir mortality may be concentrated in a specific ecoregion, peaks in Douglas-fir mortality were associated with warmer and drier conditions and often proceeded by warmer and drier conditions. Climate data from PRISM (2023).

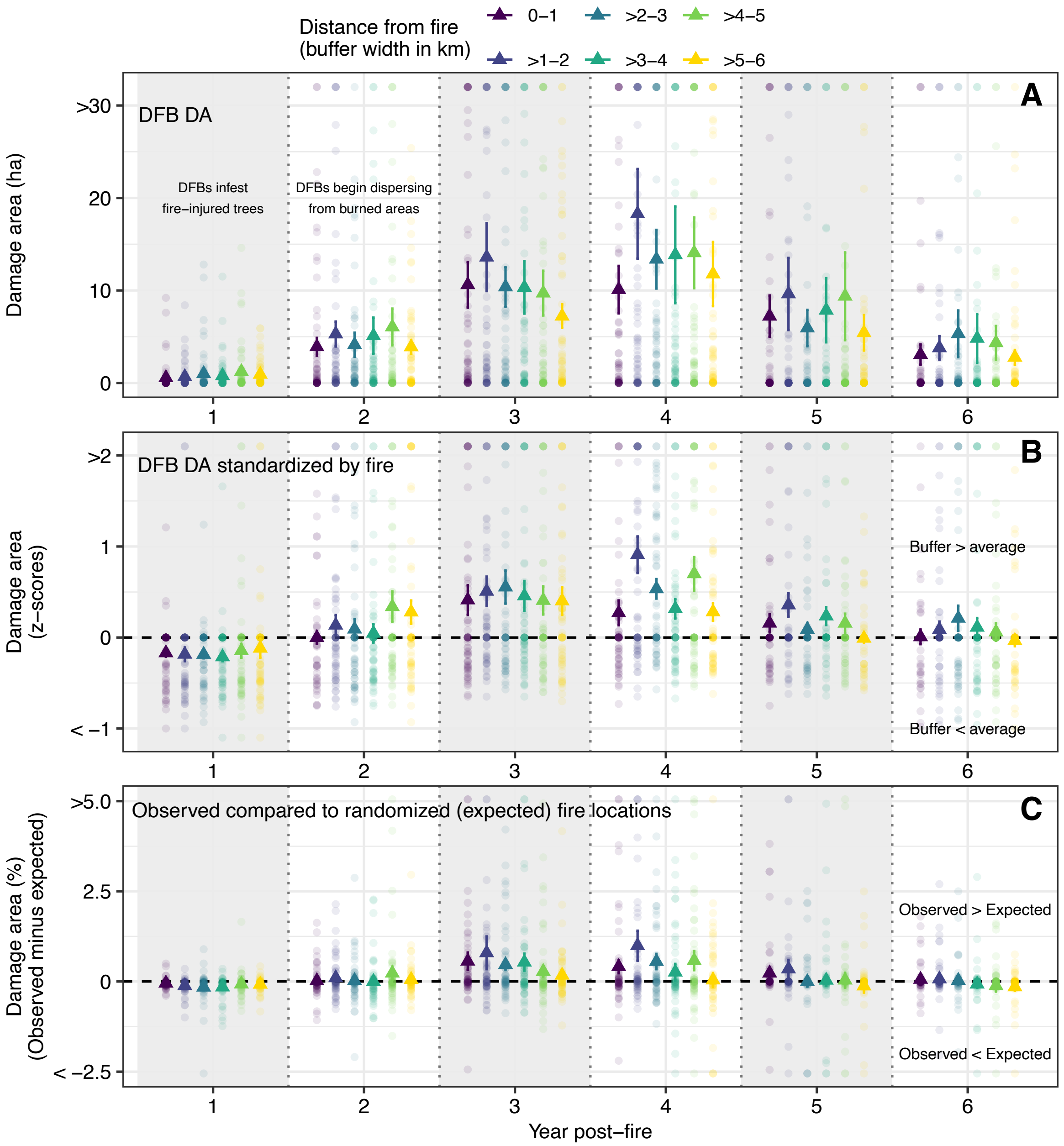

Fig. S9: (A) Average Douglas-fir (DF) damage area (DA) from Douglas-fir beetle (DFB) from one to six years post-fire for 1.0 km width buffer areas from 0 to 6 km from the fire perimeter in 61 fires in the Northern Rocky Mountains, USA, illustrating the amount and variability of DF DA surrounding fire perimeters. (B) Average DF DA with distance from fire (standardized with z-scores by fire and year) to compare DA among buffer areas within year. The expected pattern in DA resulting from DFB spillover from fire-injured trees to unburned areas (i.e., DFB spillover) would be greater DA immediately surrounding the fire perimeter (e.g., 0-1.0 km buffer areas) relative to further away (e.g., > 1 km). (C) Average of the observed DF DA (%) minus the expected DA (%) from one to six years post-fire. Observed DA was subtracted from DA in each of 15 random fire locations and then averaged by buffer and year. The vertical lines are standard errors, and the dots are values for individual fires. The same analysis was performed with buffer width of 0.5 km (rather than 1 km) in Fig. 3. In **(A)** and **(B)**, DA in the two buffer areas nearest the fire were not statistically different from buffer areas further away ($p > 0.05$).

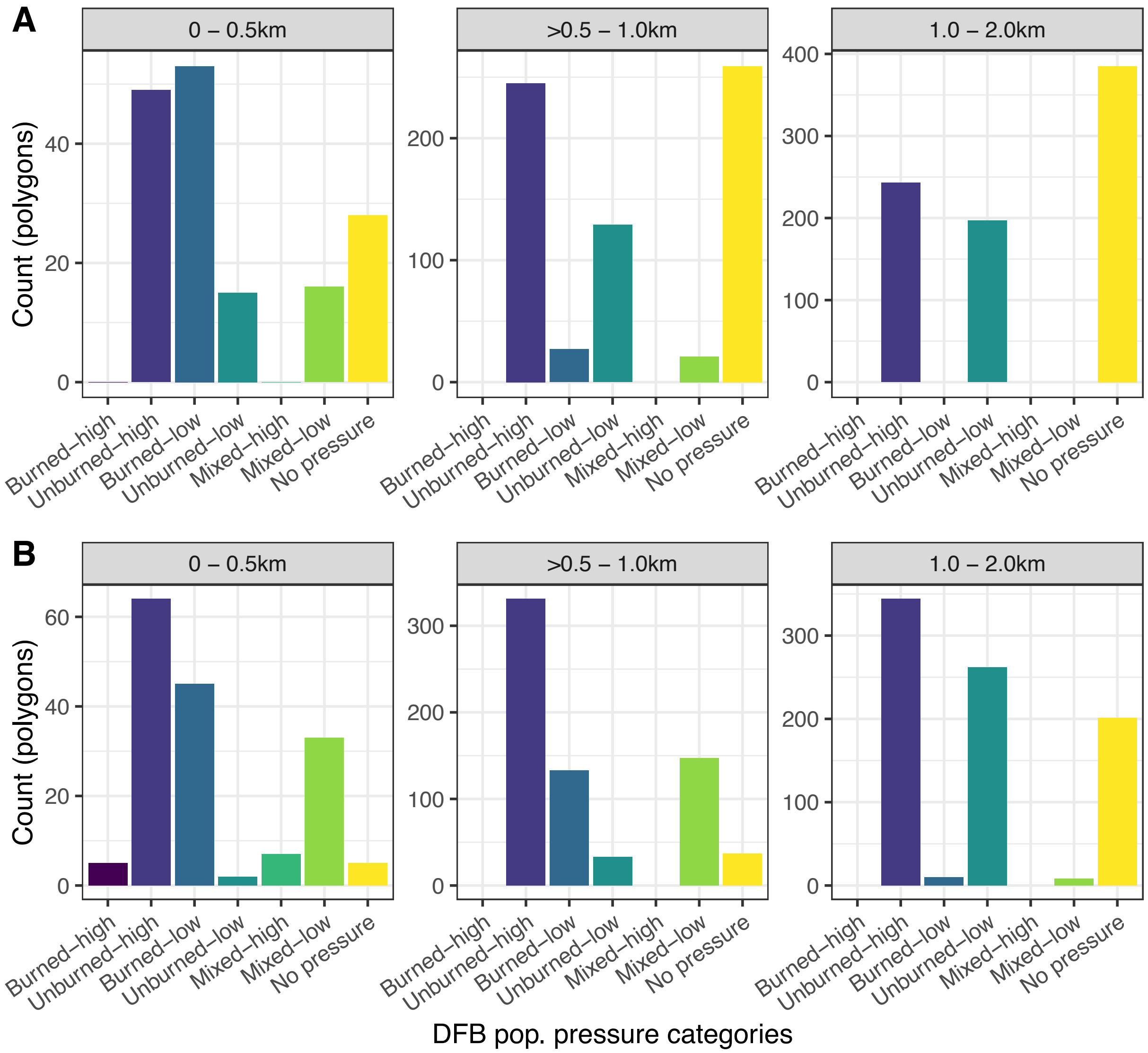

Fig. S10: Count of Insect and Disease Survey (IDS) polygons by Douglas-fir beetle (DFB) population pressure categories (x-axis) with distance from fire (panels) under the **(A)** shorter-range and **(B)** longer-range dispersal scenarios (see methods). DFB pressure categorized the size of DFB populations from burned and unburned sources (see Table S4; next page). Under both dispersal scenarios, results illustrate that DFB pressure is from unburned and burned sources < 0.5 km from the fire perimeter, with minimal DFB pressure from burned areas > 0.5 - 1 km from fire. Greater than 1 km from fire, no DFB population pressure occurred under the shorter-range scenario.

Table S4: Table of Douglas-fir beetle (DFB) population pressure categories used in Fig. S7. DFB pressure weights were 1.0 for <= 240 m, 0.3 for >240 to <=480, and 0.1 for >480 to <=720.

**Category**	**DFB pressure from unburned areas**	**DFB pressure from burned areas**
Burned high	< 0.3	= 1
Unburned high	= 1	< 0.3
Burned low	= 0	<=0.3 & > 0
Unburned low	<=0.3 & > 0	= 0
Mixed high	= 1	= 1
Mixed low	< 1 & >= 0.3	< 1 & >= 0.3
No pressure	0	0

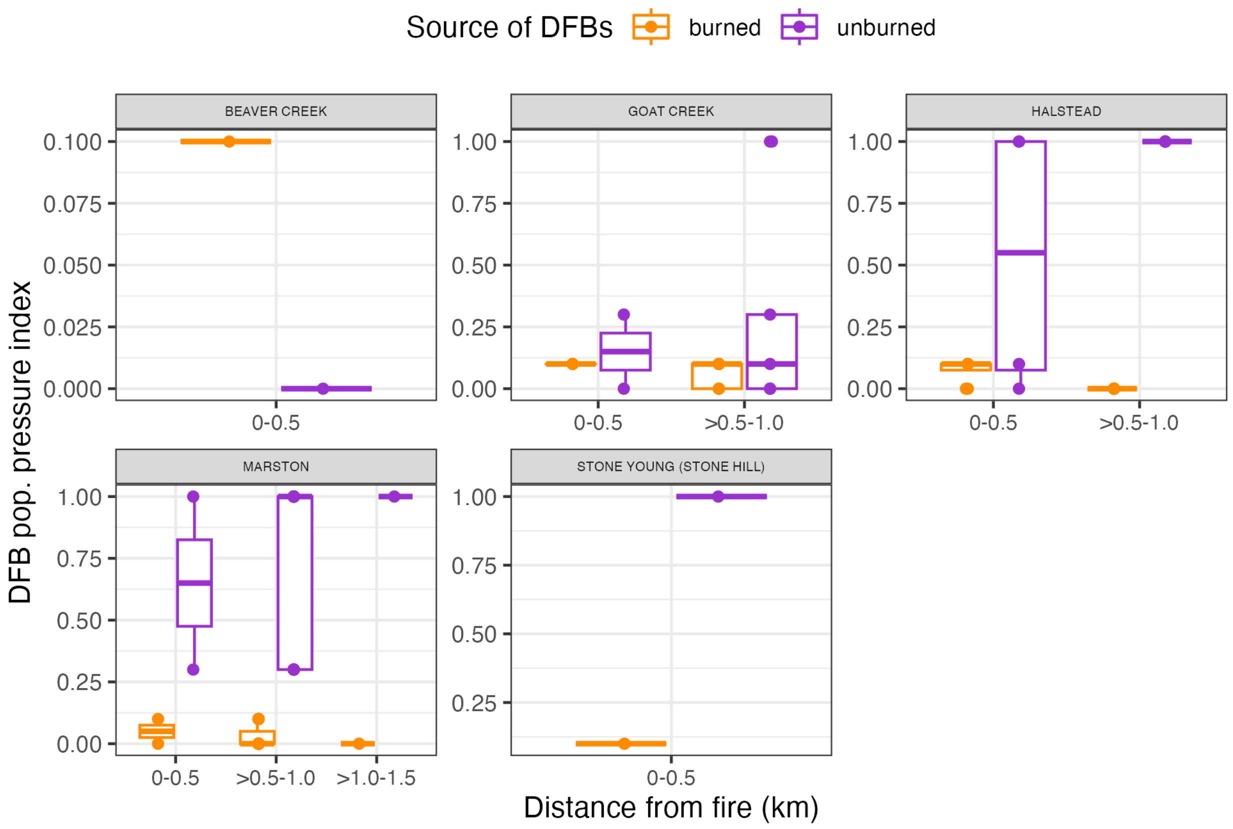

Fig. S11: Douglas-fir Beetle (DFB) dispersal pressure (shorter-range scenario) from burned and unburned sources on individual Douglas-fir (DF) mortality polygons (points in figure) mapped by Insect and Disease Surveyors (IDS) for buffer-years where DFB spillover was identified (Fig. 5). The figure illustrates that dispersal pressure on polygons from burned sources is greater than unburned sources or mixed (burned and unburned sources) for some areas < 0.5 km from fire, whereas dispersal pressure from unburned sources was generally greater than burned sources > 0.5 km (exception was the Goat Creek Fire). Results are summarized in Fig. 5B.

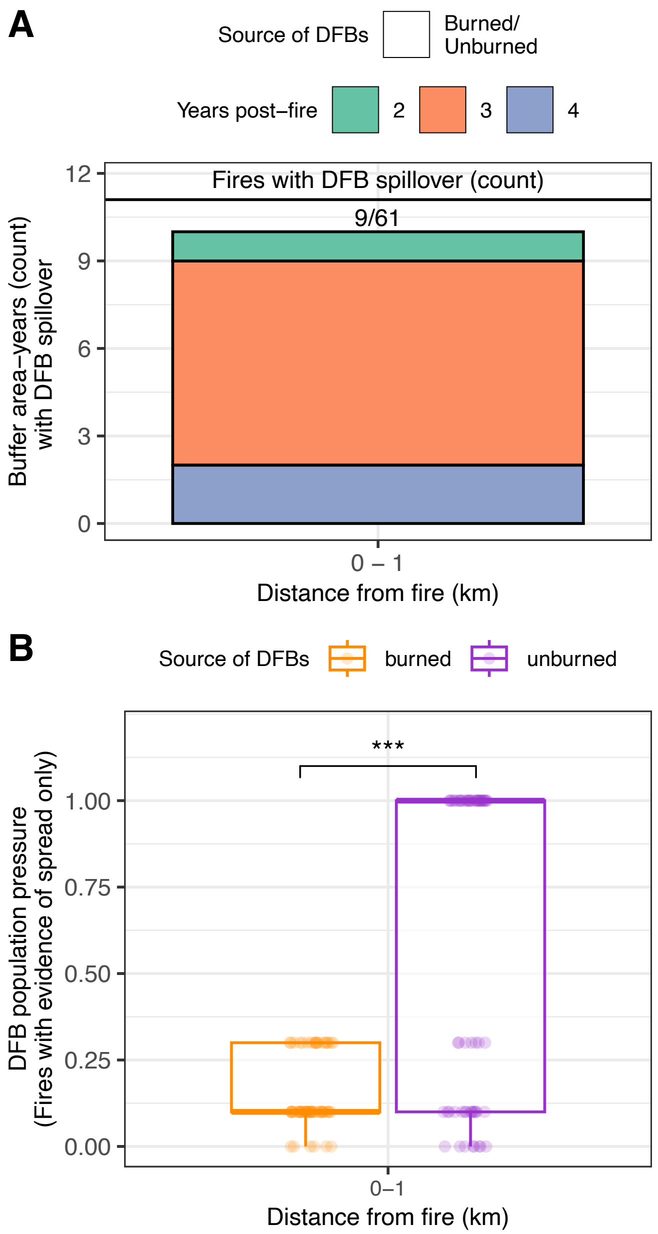

Fig. S12 (1 km width buffers): (A) Count of buffer area-years (y-axis) that met our criteria for Douglas-fir beetle (DFB) spillover, the most likely source of DFBs (burned and/or unburned; see **(B)**), and the count of unique fires with DFB spillover with time since fire and distance from fire (colors) based on the spatiotemporal progression of Douglas-fir (DF) damage area from DFB. Individual fires may have multiple buffer-years with DFB spillover. The analysis extracted DF damage area in 1 km width buffer areas from 0 to 10 km from the fire perimeter by year (i.e., buffer area-years; see Fig. 5 for 0.5 km width buffer areas). **(B)** DFB population pressure from burned and unburned sources on individual IDS polygons for fires with evidence for DFB spillover only. Within the 0-1 km buffer area, DFBs from unburned sources were statistically greater than burned sources, as confirmed by a linear mixed model ('***' $p < 0.001$). In the boxplots, the thick horizontal line is the median, the box represents the interquartile range (25th–75th percentiles; IQR) of the distribution, the whiskers extend no further than +/-1.5 times the IQR, and the dots are outliers.

Table S5: Biophysical predictor variables (one value per fire) for comparisons of fires with evidence of Douglas-fir beetle (DFB) spillover from burned areas inside to unburned areas outside the fire perimeter in Douglas-fir (DF) forest. Climate variables were extracted at the fire centroid from one to seven years post-fire.

Predictor variable	Subject	Justification
		**Climate**
Snow water equivalent (April 1)	Host	Low snowpack decreases soil moisture prior to the growing season, which may increase tree stress and susceptibility of DF to DFB attack.
Minimum winter air temperature (Dec. – Feb, ˚C)	Insect	Warmer temperatures increase rates of DFB brood development and DFB abundance (Dyer et al. 1968, Pane 2023)
May-June, May-Sept., 1-year water year (WY) 3-year WY SPEI	Host	Warmer/drier climate conditions (negative SPEI) decrease soil moisture for tree growth (Restaino et al. 2016) and host tree defenses, which may increase the likelihood for DF mortality from DFB (Powers et al. 1999). Four SPEI variables were included to account for shorter-term (May-June and May-Sept.) and longer-term (1- and 3-year water year) drought.
DRAEI		Decadal Repeat-Aridity Index: The count of years where May to September SPEI was < -1.5 in the prior 10-year period.
		**Bark beetle population**
Burned DF susceptible forest (inside fire, % of area burned)	Insect	Greater burned area of DF forest susceptible to DFB (0.5 km inside the fire perimeter) may amplify DFB populations and population pressure on areas outside the fire perimeter.
DFB DA in prior year (% of DF susceptible forest)	Insect	Greater DF damage area (DA) from DFBs in unburned areas during the prior year may increase the likelihood for DF mortality from DFB in the subsequent year in the same area.
		**Host availability and susceptibility**
DF susceptible forest (outside fire, % of area burned)	Host	Greater DF forest susceptible to DFB outside the fire (<0.5 km buffer area) may increase the host availability and the chances for DFB spread to unburned forest.
Defoliation (% of flown area)	Host	Defoliation (western spruce budworm or Douglas-fir Tussock moth) in the 2-10 years prior to DFB spillover increases likelihood for DF mortality from DFB (Cole et al. 2022; Howe et al. 2024).

Table S6: Results of generalized linear mixed model (GLMM) and generalized linear model (GLM) testing the effect of biophysical variables (see Table S5) on fires with (1) and without (0) Douglas-fir beetle (DFB) spillover. See count and year of DFB spillover fires (i.e., response variable) for 0.5 km width buffers in Fig. 5 and for 1.0 km buffers in Fig. S11. GLMM and GLM models were run separately for 0.5 km and 1.0 km width buffer areas. The GLMM was constructed for predictor variables that varied over time, including climate variables, prior year DF damage area (i.e., DFB population pressure), and prior defoliation. The GLM was constructed for predictor variables that did not vary over time, including susceptible DF forest in burned or unburned areas.

Buffer width: 0.5 km

Predictor variable	Coef.	Std error	t	*p*
**GLMM**				
No significant predictors				
**GLM**				
No significant predictors				

Buffer width: 1.0 km

Predictor variable	Coef.	Std error	t	*p*
**GLMM**				
**DF Damage area** $_{\mathbf{year-1}}$	**1.333**	**0.380**	**3.511**	**<0.01**
**GLM**				
No significant predictors				

REFERENCES

Allouche, O., A. Tsoar, and R. Kadmon. 2006. Assessing the accuracy of species distribution models: prevalence, kappa and the true skill statistic (TSS): Assessing the accuracy of distribution models. Journal of Applied Ecology 43:1223–1232.

Broxton, P., X. Zeng, and N. Dawson. 2019. Daily 4 km Gridded SWE and Snow Depth from Assimilated In-Situ and Modeled Data over the Conterminous US, Version 1. NASA National Snow and Ice Data Center Distributed Active Archive Center. https://nsidc.org/data/nsidc-0719/versions/1

Bulaon, B. M. 2003. Douglas-fir beetle surveys of the fires of 2000 in the Northern Region. Forest Health and Protection, USDA Forest Service, Missoula, MT.

Cole, H. M., R. A. Andrus, C. Butkiewicz, K. C. Rodman, O. Santiago, N. J. Tutland, A. Waupochick, and S. J. Hart. 2022. Outbreaks of Douglas-fir beetle follow western spruce budworm defoliation in the Southern Rocky Mountains, USA. Forests 13:371.

Dyer, E. D. A., J. P. Skovsgaard, and L. H. McMullen. 1968. Temperature in relation to development rates of two bark beetles. Bi-monthly Research Notes 24:15–16.

EPA. 2010. Level III ecoregions of the continental United States (revision of Omernik, 1987). Spatial (vector). https://www.epa.gov/eco-research/level-iii-and-iv-ecoregions-continental-united-states

ESRI. 2023. USA Federal Lands. Vector spatial data. https://atlas.eia.gov/datasets/5e92f2e0930848faa40480bcb4fdc44e/explore

FHP. 2023. Aerial Detection Surveys. USDA Forest Service. https://www.fs.usda.gov/science-technology/data-tools-products/fhp-mapping-reporting/detection-surveys

Hansen, M. C., P. V. Potapov, R. Moore, M. Hancher, S. A. Turubanova, A. Tyukavina, D. Thau, S. V. Stehman, S. J. Goetz, T. R. Loveland, A. Kommareddy, A. Egorov, L. Chini, C. O. Justice, and J. R. G. Townshend. 2013. High-resolution global maps of 21st-century forest cover change. Science 342:850–853.

Hood, S., and B. Bentz. 2007. Predicting postfire Douglas-fir beetle attacks and tree mortality in the northern Rocky Mountains. Canadian Journal of Forest Research 37:1058–1069.

Howe, M., Hart, S. J., & Trowbridge, A. M. 2024. Budworms, beetles and wildfire: Disturbance interactions influence the likelihood of insect-caused disturbances at a subcontinental scale. Journal of Ecology, 00, 1–18. https://doi.org/10.1111/1365-2745.14408

Krist, F. J. K., J. R. Ellenwood, M. E. Woods, A. J. McMahan, J. P. Cowardin, D. E. Ryerson, F. J. Sapio, M. O. Zweifler, and S. A. Romero. 2014. 2013-2027 National Insect and Disease Forest Risk Assessment. USDA Forest Service, Fort Collins, CO. https://www.fs.usda.gov/science-technology/data-tools-products/fhp-mapping-reporting/individual-tree-species-parameter-maps

Lazarus, L. 2011. Aerial application of MCH flakes to reduce impacts from Douglas-fir beetle on Bald Mountain Ski Area in 2010. BFO-PR, USDA Forest Service, Boise, ID.

Lowrey, L., P. Mocettini, C. Nelson, and J. McMillin. 2015. Evaluation of Douglas-fir beetle Activity in Three Burn Severities of the 2012 Halstead Fire Sawtooth and Salmon-Challis National Forests. Biological Evaluation, USDA Forest Service - Forest Health and Protection, Boise, ID.

MTBS. 2023. Monitoring Trends in Burn Severity: Burned Area Boundaries Dataset. USGS: Reston, VA, USA. https://www.mtbs.gov/direct-download

Negron, J. F. 1999. Estimating extent of mortality associated with the Douglas-fir beetle in the central and northern Rockies. Western Journal of Applied Forestry 14:121–127.

Powers, J. S., P. Sollins, M. E. Harmon, and J. A. Jones. 1999. Plant-pest interactions in time and space: A Douglas-fir bark beetle outbreak as a case study. Landscape Ecology 14:105–120.

PRISM. 2023. PRISM Climate Group. Oregon State University. http://prism.oregonstate.edu.

Restaino, C. M., D. L. Peterson, and J. Littell. 2016. Increased water deficit decreases Douglas fir growth throughout western US forests. Proceedings of the National Academy of Sciences 113:9557–9562.

Ryan, K. C., and G. Amman. 1996. Bark beetle activity and delayed tree mortality in the Greater Yellowstone Area following the 1988 Fires. Pages 151–158 Ecological implications of fire in Greater Yellowstone Proceedings. R.E. Keane, K.C. Ryan, S.W. Running (eds), Fairland, WA.

Shaw, J. D. 2000. application of stand density index to irregularly structured stands. Western Journal of Applied Forestry 15:40–42.

Speer, J. H. 2010. Fundamentals of tree-ring research. Univ. of Arizona Press, Tucson, AZ.

USDA. 2022a. Timber harvest. USDA Forest Service. http://data.fs.usda.gov/geodata/edw/datasets.php

USDA. 2022b. Hazardous fuel treatments. USDA Forest Service. http://data.fs.usda.gov/geodata/edw/datasets.php

USDA. 2022c. Silviculture timber stand improvement. USDA Forest Service. http://data.fs.usda.gov/geodata/edw/datasets.php

Weatherby, J. C., P. Mocettini, and B. R. Gardner. 1994. Biological evaluation of tree survivorship within the Lowman Fire Boundary, 1989-1993. Page 9. USDA Forest Service, Forest Health Protection, Boise Field Office, Boise, ID.
